# Prize Hospital Report, from St. Bartholomew's Hospital. (Prize Adjudged to Mr. Fereday, Dresser.) No. III

**Published:** 1827-10

**Authors:** 


### PRIZE HOSPITAL REPORT,

### *No.* III.

Mr. FEREDAY,

BARTHOLOMEW'S HOSPITAL.

### CASES OF PHLEBITIS.

Mr. Abernethy* has observed, that he has not seen an instance of inflammation of the vein, consequent on venesection, which proceeded to suppuration. Such diseases must, consequently, be rare; and it is singular that no less than three cases should have been admitted into this hospital during the space of two months, under the care of Mr. Lawrence. The following is a detailed account of them.

*Case* 1. Sophia Branclin, æt. 25, had married at the age of 15, and, since that time had been in the habit of drinking freely,—more particularly spirits. Whilst crossing a street, six days ago, she was knocked down by a cart, and, on account of some bruises which she received, was bled next day, from the left median basilic vein. She kept her arm in a sling the remainder of the day, and returned to her usual occupation of weaving the following morning, which required very considerable exertion of her arm. Towards evening, the arm felt stiff and painful, and the bandage tight. Next day (Wednesday) she continued working, which produced a sensible aggravation of all the symptoms; and on Thursday and Friday was incapable of using the arm, which she poulticed; and, as she felt herself very unwell, came to the hospital the following morning.

She was admitted the 2d December, when the following symptoms were observed:—The arm, for some distance above and below the elbow-joint, is swelled, hard, red, and very painful on pressure. The wound in the vein has a small crust over it. The face is pale and anxious; the skin hot and dry; pulse full, incompressible, and 120; tongue white and moist; great thirst, no appetite; bowels well open, from some medicines she took the day before.

V.S. ad ℥xvj. vel ℥xx. hirudines xx. brachio, vespere. Fomentatio contr. brachio appl. Cal. gr. iv. jalapæ, gr. xij. statim sum. Mistura ammon. acetatis, (liquoris ammonia acet. ℥ij. aquæ, ℥vj.) cum liq. ant. tartarizati, ʒss. sextis horis sum.

She fainted when sixteen ounces of blood had flowed, which was much cupped and buffed

*December* 3*d*. In the evening she felt relieved, and was less feverish. She passed a very restless night, and has slept very little, owing to the pain in her arm. The skin is very hot; respiration hurried, and she moans a gréat deal; pulse small, and 140; bowels open. She complains of pain in the abdomen, which is increased on pressure, and on a deep inspiration, which she supposes proceeds from the hurt she received from the fall, for which she was first bled.

Rep. hirudines xxx. brachio; et postea cataplasm. farin.

4*th*. Has had a better night, and feels easier this morning. The pulse is less frequent, and the arm is less painful; complains greatly of the epigastric region.

Hirudines xxx. epigastrio. Mist. ammon. acet. cum liq. antim. tart. ʒss. octavis horis sumend. Cal. gr. iij. quartis horis sumend.

5*th*. Has slept very little, and continues moaning, without complaining of any pain. The inflammation of the arm extends towards the axilla; the pain in the

* Surgical Works, vol. ii. page 151.

epigastrium is quite gone since the application of the leeches. The tongue is white on the sides; light brown and dry in the middle. In the evening she complained of great pain in the limbs, particularly of the left knee-joint.

*6th.* Continues much the same; but her countenance is now pale, mixed with a tinge of yellow, and very anxious. She complains of soreness of the mouth; the inner side of the lips, &c. are covered with aphthæ.

*7th.* Continued very restless during the night, and got no sleep; the arm is less inflamed, and the wound in the vein discharges rather copiously; the pus, which is of the ordinary consistence, is sometimes of the usual colour, sometimes red, from the admixture of blood. A considerable quantity of this red matter issued from the vein to-day on pressure in the neighbourhood of the wound. Pulse small, and 104; tongue furred, and dry in the middle; bowels open. She complains of pain over all the body, which is particularly severe in the calves of the legs, so as to make her shrink when moved.

*8th.* Continued restlessness and want of sleep; countenance flushed and anxious; respiration hurried; the pains of the limbs, particularly of the calves, continue; the redness, heat, and tension of the left fore-arm rather increased; the purulent discharge from the wound continues; the tongue is less dry; the bowels have been kept continually open by the calomel, which seems beginning to affect her mouth.

Hirudines xxx. brachio. Cont. fomentatio. Int. cal. et mistura ammoniæ acetatis.

*9th.* Has been very restless during the night. Her countenance is more anxious and sallow, with a yellowish tint; the arm is better; tongue dry and brown; considerable thirst; pulse full, soft, and 100; bowels open.

Mist. ammon. acet. ℥ij. Vini sem. colchici, ʒj. ter quotidie sumend. Tinct. hyosciami, ʒj. horâ somni, et rep. si opus sit.

*Evening.* A dose of colchicum and hyosciamus has been taken. The pulse is full and frequent; the skin warm, but moist; the tongue moist; the pains in the limbs diminished, and she feels better.

*10th.* Had some refreshing sleep, and is in all respects better to-day. The swelling and pain of the arm are nearly gone; thin pus still flows freely from the orifice of the arm.

*11th.* She slept a little in the evening, but was restless during the night, and complained much of the pains in her limbs. Her appearance is improved; the left arm is nearly of the natural size; respiration nearly natural; pulse full, soft, and 108; the tongue dry, and a little brown in the middle; thirst diminished; bowels purged.

*Ten* P. M. She has a paler and more anxious countenance than in the morning; the pains in the limbs, however, are much diminished. Skin is now cold; respiration laborious, and thirty in a minute; pulse small, weak, and 104. Bowels continue purged, and the abdomen painful on pressure.

*12th.* Has passed a more quiet night, having slept for five hours. Her countenance much altered and pale. Matter has formed under the skin of the right fore-arm, without any previous redness: two ounces of good pus evacuated by a puncture. At two other points in the same fore-arm there was an appearance of fluid presenting, but also without discoloration of integument. Painful swelling of the left knee, from effusion into the articular cavity. In the evening, her appearance was more ghastly, and her respiration was so loborious as to require her head being raised

Cal. gr. x; opii gr. j. statim. et bis die rep.

*13th.* Only one dose of the colomel and opium has been taken; it caused profuse purging. She has passed a very bad night, and is much worse to-day. Her appearance is cadaverous; her eyes have lost their usual animation; respiration very laborious, and forty; pulse small, weak, and 100; mouth parched; thirst; bowels purged.

Intr. cal. et opii.—Mist. cretæ comp. ℥j. c. tinct. opii, gtt. xx. statim, et si opus sit repet.

*14th.* Died early in the morning.

The body was examined thirteen hours after death. The husband chose to be present at the examination, and to restrict it in point of time and extent; it was therefore hurried and imperfect. The following points were ascertained:—inflammatory condensation of the cellular tissue of the fore-arm and arm in the inflamed part. A chain of small suppurations in the course of the blood-vessels, with white healthy pus, from below the elbow to the axilla. The basilic vein, being slit open to the axilla, was found filled with pus to the point where it joined the axillary vein, where a large coagulum, not adhering very intimately to the coats, was situated. To this point the internal surface of the basilic vein was thickened, had a rough appearance, and was of a pale yellowish colour. Beyond the coagulum in the axillary vein, other coagula were found, of a more recent formation, filling up most of the larger veins and the cavity of the heart. In other respects these vessels appeared quite natural. No diseased appearance in any of the other organs of the chest. The liver light coloured, and beginning to assume the yellow appearance produced by indulgence in spirits. The other abdominal viscera appeared sound. The mucous membrane was not examined. Neither the head nor knee-joint were allowed to be opened.

*Case* 2. John Carr, a strong healthy man, aged forty-seven, whilst following his occupation of smith, "strained his back," which gave him great pain. He continued working about a week, when, finding he got worse, he came to the hospital on the 2nd of January. He was bled from the median basilic vein, and experienced considerable relief at the time. Next day he returned, having been very feverish during the night, with pain in the wound of the vein, and inflammation of the surrounding cellular tissue. His face is pale and anxious; his chest painful, especially on taking a long inspiration; and his pulse full, hard, and frequent.

Hirudines xxx. brachio, et postea cataplasma panis.—Liquor. antimonii tart. ʒss. quartis horis sumend.

*January 3rd.* Has passed a good night, and feels better this morning. The arm continues inflamed, and very painful on pressure. V. S. ad ℥xx. The blood is buffed and cupped, and the serum has a milky appearance.

Hirudines xx. brachio vespere, cal. gr. iv. jalapæ, gr. xij. statim.

No sensible effect produced by the antimony.

He was better in the evening; his pulmonary complaints are much relieved; and his bowels are now freely opened.

*4th.* Continues better. Is still purged; pulse full, hard, and 106; thirst. Dr. Wise examined the chest with the stethoscope, and found the lungs every where pervious to air; the action of the heart was distinct over a larger space than natural, and the movements of the right ventricle were strong.

Empl. lyttæ, sterno.—Mist. ammon. acet. ℥ij. 8vâ quâque horâ sumend.—Cal. gr. iv. ter die.—Venesectio ad deliquium. Syncope was produced when 20 oz. had flowed. The blood was highly cupped and buffed. He is now much purged. Int. liq. ant. tart.

*5th.* The swelling of the arm is increased, and the discharge from the wound considerable. The symptoms connected with the thoracic affection are nearly gone. In the evening, he had several shivering fits, and he appeared low and irritable.

Hirudines xij. brachio.

*6th.* Continues to sleep well. His countenance is very sallow; he is in good spirits; has no pain; pulse small, soft, and 140. The nervous irritability, which has been observed for several days, is increased this evening.

*7th.* He has been very thirsty during the night, and complains much of weakness this morning. Respiration natural; pulse full, soft, and 112; bowels open. There is still a hardness along the course of the inflamed vein; the redness is gone, and he has no pain. He will not take his medicines.

*10th.* Continues much the same; his face is paler, and it has a yellowish tinge. Respiration thirty-two, without any pain; pulse small, and 108; tongue slightly brown and dry; thirst; bowels open.

*12th.* Is very restless and low; says he has no pain, but that he is quite well if he had strength. The inflammation in the arm almost entirely gone near the elbow-joint, but extends towards the axilla. A considerable quantity of fetid pus, mixed with air, can be pressed from the wound in the vein.

Mist. sennæ comp. ℥iss. (Infus. sennæ comp. ℥vijss. tinct. sennæ ℥ss. magnes. sulph. ℥iss. ♏ ft. mist.) Cont. mist. ammon. et cataplasma panis ut antea.—Tincturæ hyoscyami, ʒj. horâ somni.

13*th.* The inflammation in the arm is extending towards the axilla, whilst it is much diminished at the bend of the arm.

14*th.* On the inner side of the insertion of the deltoid muscle a considerable swelling appeared, with a feel of fluctuation, without any surrounding inflammation.

15*th.* His countenance is still yellowish, and he complains of his great weakness.

17*th.* Is very restless during the night, and gets little sleep; he is more feverish this morning; great thirst; and moans much.

Mist. ammon. acet.; tinct. digitalis gtt. xv. 8vis horis sumend. empl. lyttæ sterno.

18*th.* Continues restless, and is very irritable this morning, which is particularly evident in coughing, when his whole body is violently shaken. The cough is dry, attended with much pain. Pulse full, soft, 86; tongue moist and brown; no thirst. There is very little discharge from the wound in the vein. The small tumour over the vein, near the insertion of the deltoid muscle, has still the natural colour of the skin; but is soft, and, as it now evidently contains a fluid, it was punctured, and a very thick, adhesive, yellowish, fetid pus was discharged. Will not take any of the medicines.

19*th.* Last night and to-day he complains of the pain in his side.

20*th.* Coughed a good deal during the night, which gave him great pain. He continues very irritable, and coughs a good deal, which is now accompanied with copious purulent expectoration. Pulse small, soft, and 104; thirst; bowels open.

23*rd.* The cough is a little better, and the pain in the chest is not so violent. Has no appetite; bowels open; the wound in the vein has ceased to discharge.

25*th.* Last night, after having drank some cold fluid, he had a severe shivering fit, and during the night had several more. He is very low this morning, and complains much of weakness. Pain on pressure over the seat of the liver; cough diminished; the arm is nearly the natural size and appearance.

27*th.* During the night he was very restless and irritable, and several times endeavoured to get out of bed. The weakness seems to be much increased, and he complains much of pain in the right arm. Respiration sonorous, and 68; pulse full, soft, and 120. Great thirst; cough diminished; tongue dry and brown; bowels open.

28*th.* Has been delirious during the night; the weakness and irritability are increased; pulse full, soft, and 136; pressure over the seat of the liver gives him pain.

29*th.* Continues very irritable, and speaks a good deal incoherently. This morning he appears sinking; his countenance has a pale yellowish appearance; respiration sonorous, and sixty-six; bowels well open.

30*th.* Great difficulty of breathing; has frequent attacks of a painful shivering fit, between which he dozes a little. He is retained with difficulty in bed; his tongue is dry and covered with a brown coat. Towards the evening, the difficulty of breathing and shivering fits increased.

He continued in much the same state until six o'clock the following morning, when he expired.

*Dissection,* eight hours after death. The basilic vein was exposed from the middle of the fore-arm to the axilla. A small abscess, with a smooth secreting surface, was found opposite the external wound. The median basilic and the basilic veins, for about two inches, were impervious, and reduced to a cord-like substance, like the umbilical vessels in young subjects. On following the basilic vein above this impervious portion, it was found to terminate in an oblong-shaped abscess, about two inches long, which communicated above with the basilic vein. The surface of this abscess was lined by a smooth secreting membrane: it was near the middle of this abscess that the opening had been made during life. The basilic vein beyond this abscess was rough, and covered with a false membrane for about an inch, when it terminated in a conical shaped portion of fibrin, which filled it up and adhered firmly to the coats of the vein. Immediately above this, the veins appeared healthy, where several considerable vessels poured in their blood. Recent coagula filled the other large veins and the cavities of the heart, which appeared healthy.

A few ounces of yellowish serum, mixed with flocculi, were found in the pericardium; and several large white spots were found on the surface of the heart.

Extensive recent adhesions were found between the pleura costalis and pulmonalis. Several vomicæ were found in the lungs, which otherwise appeared healthy. A large quantity of mucus, mixed with air, was found in the bronchia.

Extensive recent adhesions were found between the upper surface of the liver and diaphragm.

The scull-cap adhered firmly to the dura mater; and, on separating them, some serum escaped. The arachnoid membrane was thicker, more opaque than natural, and was elevated by effusion of a straw-coloured fluid, mixed with serum under it; which was peculiarly evident at its posterior part. On dividing the hemispheres of the cerebrum on a level with the corpus callosum, an unusual number of bloody points were found in its substance. The lining of the ventricles was particularly evident, and they contained several ounces of a yellowish serum. The substance of the brain had its usual hardness.

*Case* 3. Henry Arnold, a very stout porter, fifty-one years of age, was admitted into the hospital on the 19th of January, on account of an old ulcer in the leg; which, from walking about and intemperate habits, was then very much inflamed. Soon after admission, (with aperients, &c.) he was twice bled from the arm. After the second venesection, performed in the left cephalic vein, being very restless, the bandage was twice loosened, and he lost a quantity of blood each time.

On the 26th of January, three days after the last bleeding, he complained of the bandage on the arm being tight. It was removed, and the edges of the wound in the vein were found slightly inflamed.

28*th*. The arm felt easy till to-day, when he again complained of pain; a slight redness surrounds the wound, which is open. The arm was poulticed, and it felt better till the evening of the 29*th*, when he had several shivering fits, followed by a state of feverishness.

30*th*. He appears pale this morning; his pulse is small, hard, and 120; thirst; no appetite—tongue white and moist—bowels open. The arm is swelled, particularly along the course of the cephalic vein, where the skin is red, and very sensible to the touch. The wound in the vein remains open, but there is no discharge.

R. Cal. gr. iv. Jalapæ, gr. xij. statim. V. S. ad ℥xvj. Liq. ant. tart. ℥ss; potassæ nitr. ʒss. quartis horis sumend: Hirudines xx. brachio, vespere.

31*st*. The arm is less inflamed this morning, and he seems better. The antimony produced great sickness, and most distressing retching.

Cont. Liq. ant. tart. ʒij; potassæ nitr. ʒss. quartis horis. Hirudines xxx. brachio et postea emplastrum lyttæ amplum.

*Feb.* 1*st*. The pain arising from the blister has prevented him from sleeping much during the night. The antimony produced distressing sickness and vomiting: to be discontinued. Pulse small, and 102—has no appetite—tongue white—bowels open.

2*nd*. Has slept better last night, and this morning is easier. His face has a yellowish appearance; his respiration sonorous, and twenty-six—pulse small, soft, and 124—tongue clean.

3*rd*. The pain along the course of the cephalic vein prevented him sleeping in the night. A little healthy pus was pressed out through the wound in the vein. In other respects he appears to be in much the same state as yesterday.

R. Mist. ammoniæ acetatis, ℥ij. tinct. digitalis, gtt. xij. quartis horis sumend: Cal. gr. iv. sextis horis sumend.

4*th*. Had a shivering fit yesterday afternoon, but in the evening was better. He then complained of pain in the knee-joint, which increased during the night so as to prevent him sleeping. The knee-joint and thigh are very much swollen, and very sensible on the least movement.

5*th*. Has had a better night, and his knee and thigh feel easier. The hardness and swelling of the arm are almost gone; it is not sensible on being pressed, and the discharge of pus from the wound in the vein has ceased. The cavity of the left knee-joint is distended with fluid, causing a prominent tumefaction above the patella; all the superficial veins are turgid, and form a network under the skin. Pulse full, soft, and 140; great thirst; tongue white; bowels open. Has taken four doses of digitalis, and six of calomel.

Cucurb: cruent. ad ℥xvj. genu. Cont. tinct. digitalis et cal.

*6th.* Has been very restless during the night, and complains of great pain in the right shoulder. The left knee and thigh have been easier since the cupping, but appear to be much in the same state as yesterday. Respiration laborious, and thirty-four; pulse hard, full, and 126; tongue white on the edges, and brown in the middle—great thirst—bowels not well open.

Mist. sennæ comp. ℥ij. statim.

*7th.* Has been delirious during the night; his bowels are well open, and this morning the stools have passed involuntarily. He is much lower. The left lower extremity is more swelled, particularly round the knee-joint, but he does not complain so much of the pain. Breathing laborious, and forty-two; pulse small, and 120—great thirst—tongue brown and dry. He continued much in the same state till early next morning, when he expired.

*Dissection, ten hours after death.* The cellular substance of the arm was thickened, and about its middle an abscess was cut into. The cephalic vein was found to contain a considerable quantity of healthy pus, for about two inches below, and four above the wound in the vein. Beyond these points coagula of blood were found, separating the diseased from the healthy portion of vein. The internal surface of the portion of vein filled with pus was irregular and thickened. The larger vessels appeared healthy. On the surface of the heart a few white spots were observed.

The arachnoid membrane was of a whitish colour, more opaque than natural, and under it a considerable effusion of serum was found in the reticulated substance of the pia mater. The ventricles contained several ounces of serum. The knee-joint contained a large quantity of pus, discoloured by the admixture of blood. The synovial membrane was thickened, and very vascular. Considerable absorption of the cartilaginous surfaces had taken place; the extremities of the femoral condyles, and the corresponding extremities of the tibia, were completely bare.

The cellular membrane covering the capsule of the joint, under the extensor muscles, was inflamed, thickened, and loaded with pus; it was in the same state throughout the whole substance of the vasti and cruralis muscles, and on their surface. Sections of these muscles presented their fasciculi, separated apparently by layers of thick yellow pus. The matter was no where collected into an abscess, but diffused throughout the cellular texture as serum is in the case of anasarca. In the rest of the limb, the cellular tissue was filled with a bright and light yellow serum. The cellular substance on the outside of the orbicular ligament of the right shoulder was filled with pus; but the cavity of the joint, and the deltoid muscles, were natural.

*Remarks.* It has been supposed that phlebitis after bleeding, is produced by the employment of lancets or sponges bedewed with animal matter. In all the above cases, the lancets, as far as could be ascertained, were perfectly clean; and sponges were not employed, but lint.

In two of the cases, inflammation seemed to have been produced by motion of the arm too soon after bleeding. In the third case, the inflammation might be connected in part with a certain predisposition to the disease, which inflammation of the serous membrane may give; in the same manner as phlebitis frequently gives rise, during its course, to pleuritis, &c. This latter circumstance, the frequent occurrence of inflammation of several membranes supervening on phlebitis, is illustrated by the above case, and by other fatal cases of the disease which have been communicated to me. And it is still further supported by experiments upon animals, which Dr. Wise informs me he made whilst investigating the same subject.*

There are some points of similarity in the above cases: in all, the local symptoms were followed by severe inflammatory fever, and inflammation of a serous membrane. As the disease advanced, pus

---

* Some of these experiments were the repetition of those of other enquirers. See Sasse de Vasorum Sang Inflammatione, Halle; and Gaspard, Journal de Physiologie, tome 2, p. 7.

was discharged by the wound in the vein or by abscesses in the cellular lesion.

As the symptoms of the internal inflammation advanced, that in the vein proceeded more slowly. The inflammatory fever was followed by symptoms of depression, which generally resembled very much those of the epidemic fever of this country.

The examination after death proved, that the local effects of the bleeding were an inflammation of the vein, and of the surrounding cellular substance; and in consequence of which, either from the structure or function of the part inflamed, inflammation of other and distant parts followed, attended with much danger to life. Such effects may be compared to fever accompanied with inflammation of the mucous membrane of the intestines, or syphilis with affection of the throat, &c.

In the treatment, our first endeavour ought to be directed to the diminution of the local inflammation by bleeding, general and local, and the other parts of the antiphlogistic treatment and regimen.

When the inflammatory fever is severe, the most powerful antiphlogistic remedies ought to be employed, as large general bleeding; after which grain doses of the tartrate of antimony, repeated every two or four hours, and its action kept up for a considerable time. By following this plan of treatment, I have known two very severe cases of phlebitis cured. It may become a question, seeing the frequent fatality of the disease, whether it might not be advisable to adopt the plan of dividing the vein transversely, as high up the arm as possible, and that at an early stage of the disease. By dividing the vein, the continuity of surface between the diseased parts and the heart is destroyed, when its effects will not be much different, most probably, from those of a common abscess. Even when the constitutional symptoms have been present for a considerable time, we ought to divide the diseased vein as high up as possible; by which the local cause will not keep up the constitutional effect.

Provided the inflammation exist to an extent only capable of producing the adhesive process, a slight degree of pressure on the vein above the wound, would probably cause the adhesion of its sides; and, by preventing the access of pus (which might subsequently be secreted) into the general circulating mass, cut short the progress of the disease; leaving only an impervious state of the vessel.

A gentleman from the United States informs me, that a very successful practice adopted in that quarter of the globe, consists in the application of a large blister to the affected part in the early stage of the disease, extending, in either direction, three or four inches beyond the inflamed vessel. It was first recommended by Dr. Physick of Philadelphia.

In the latter stage of phlebitis, when the patient appears very low, tonics have been given, but generally with the worst effect. The depressed state of the system in this case seems to be produced by the severity of the inflammatory disease, which is rapidly increased by the injudicious use of tonics.

These indications of cure must be modified by the peculiar circumstances of the case, which must be left to the judgment of the practitioner.

Information upon rare and fatal cases is valuable, as tending to elucidate the subject; I therefore relate the following.

---

CASE OF TRAUMATIC TETANUS, SUPERVENING A SLIGHT ULCERATED WOUND OF THE FORE-FINGER.

John Cooper, æt. 32; admitted on the 22d of May, under the care of Mr. Earle. The account received from this patient is, that on the 15th May, he received a slight wound on the fore-finger of his right hand from the falling of the tail-board of his cart. He treated it very lightly, and pursued his accustomed labour until yesterday morning (21*st*) when he experienced a slight shivering, together with a degree of stiffness about the back of the neck, which rendered the motions of the head painful and difficult. Conceiving these symptoms arose only from a slight cold, he went out as usual, but returned in the afternoon exceedingly unwell. His wife was sent for about four o'clock, who found him in bed lying on his belly, and unable to turn. He complained of great pain in his back and neck, more particularly in the throat. At this time there was no trismus. The symptoms alarmed her, and she sent for medical assistance. At 8 P. M. a medical gentleman saw him, who enquired of the patient if he had received any injury. At first he was answered that he had not; but, upon recollecting himself, informed him of the slight injury he had received on the finger. The wound was examined, and found as the patient had described it, nearly well. The pulse at this time was full and hard; the spasms, which occurred about every half hour, were very severe, and there was some degree of trismus. He was immediately bled from the arm to ℥xxxvj. which produced syncope. Hydr. submur. gr. iv. opii gr. j. 2ndâ. vel 3tiâ, quâque horâ.

The bleeding afforded some relief, feeling somewhat easier about his back and throat.

His medical attendant again saw him the next morning. He had passed a very restless night, and was evidently much worse. The spasms recurred every fifteen minutes; pulse hard and 120; trismus to a considerable degree; bowels not opened since yesterday evening. He was sent to the hospital at half-past twelve o'clock P. M., when he was found labouring under the following symptoms:—Muscles of the back and neck very rigid; pain at the root of the tongue; much difficulty in, and aversion to, swallowing; some degree of trismus; countenance expressive of very great anxiety; respiration hurried and difficult; skin, contrary to its usual state in these cases, hot and dry. Previous to the return of the spasm, he complains of a severe constrictive pain, which he refers to the bottom of the sternum, darting backward from this point to the spine, evidently in the direction of the diaphragm, which muscle is immediately afterwards convulsed, and subsequently the muscles of the back and trunk. These increasing at times produced opisthotonos. Pulse full, hard, and 120; tongue could not be examined.

Hydrargyri submuriatis, gr. iij. jalapæ, gr. xij.; 2dâ quâque horâ. Misturæ sennæ comp. ℥viij. olei ricini sp. terebinthinæ, aa ℥j. M ft. enema, statim injiciendum.

*Two o'clock* A. M. Spasms more frequent; complains of pain all over his body.

C. C. ad ℥xl. nuchæ et dorso.

Was convulsed three times during the operation. The cupping has had a decided effect; the pulse is quick and fluttering; the whole body in a profuse cold perspiration; spasms not so frequent; says he feels a little better; bowels obstinately costive. The catheter was introduced, and about a quart of high-coloured urine drawn off.

Cont. cal. et jalap. omni horâ—Rep. enema.

*Four o'clock* A. M. The spasms have returned with renewed force; has great aversion to take his medicines; complains greatly of his throat, and feels as if he should be suffocated; the mental faculties are entire; bowels not opened; pulse small, irregular, and 140.

Cont. cal. et jalap.—Rep. enema.

*Five o'clock* A. M. Spasms general and violent; pulse irregular, and not to be counted; respiration greatly affected; is convulsed every five minutes; bowels have not been opened. He lingered until six o'clock, and then expired in a convulsion which lasted two minutes.

*Sectio cadaveris—24 hours after death.*—The dura-mater was healthy, and a quantity of serum was found between the arachnoid membrane and pia-mater. The substance of the brain was more vascular than usual, and there was more fluid in the lateral ventricles than natural.

That portion of the spinal cord between the third and fifth cervical vertebræ was highly injected; but of its usual consistence. The remaining portion was perfectly healthy.

The sympathetic nerve was very carefully examined, but nothing unusual was observed.

The mucous membrane lining the œsophagus, cardiac portion of the stomach, larynx, and bronchial tubes, was very vascular.

The heart and lungs were perfectly healthy; the blood found in the former was quite fluid.

The abdominal viscera were sound; the intestines inflated with gas; and the rectum was loaded with fæcal matter. The wound in the finger, which was not, originally, more than a quarter of an inch in extent, and which had completely cicatrized, was carefully examined, and found not to extend deeper than the integuments. It had not reached any nerve of importance, nor injured any tendon.

The radial and median nerves which supply the finger were carefully dissected; their branches were found entire, and were not in the slighest degree inflamed.

---

EXCISION OF A POLYPUS FROM THE UTERUS.

Susan Squires, æt. 48, became a patient in this hospital on the 26th of February, under the care of Mr. Earle. She has been married nine years, previous to which time her health has always been good, and the uterine functions performed with great regularity. Eighteen months after marriage she was put to bed, after a protracted labour, of a dead child. About two years and a half afterwards, she was again confined, and had an easy labour. She suckled the child a year and a half, and when it quitted the breast suffered from profuse and constant floodings, which continued, more or less, for two years and a half, during which time she was treated as a case of common menorrhagia. After her health had suffered considerably she submitted to an examination, when a large polypus was discovered. She was then directed to come to this institution, where an examination detected a polypus filling the whole of the vagina; attended with a considerable discharge of thin bloody serum. Her health and strength were considerably reduced. It was determined to remove the polypus by ligature in the course of a day or two; but from some cause or other the operation was postponed; and, during this delay, it had descended to such an extent that the greater bulk of the tumour had protruded through the os externum, causing considerable pain by the dragging down of the uterus. Its size was now distinctly ascertained to be about equal to that of a child's head at birth; and it was found to be growing from the cervix uteri; for, when it had descended to its greatest extent, the cervix was distinctly observed giving origin to it. Mr. Worry, one of the house-surgeons, was called to her in the night, and found the poor patient in a complete state of syncope, from the loss of blood she had sustained. He conceived that the only remaining hope of saving her was immediately to remove the polypus by excision; and accordingly placing a ligature very tightly around its neck, detached it immediately below with a scalpel. Very little bleeding followed. Eighty drops of laudanum were administered in a little wine. The patient soon felt easy. It would be needless to give daily, or detailed accounts of the further progress of the case, as they would contain nothing but repetitions of her continued favourable progress. The ligature came away in about a fortnight; the floodings were suppressed; the pain ceased; she rapidly acquired her former health and strength; and was discharged, quite well, on the 3rd of April.

---

FRACTURED RIBS WITH EMPHYSEMA.

*Case* 1. Joseph Brooks, æt. 37, was received into the hospital under the care of Mr. Earle, about seven o'clock on the evening of the 20th of March, having, about two hours before, fallen from the shaft-horse of a dray, the broad wheel of which passed over his chest. He was taken up by some men, and conveyed to an adjoining house in an exhausted and apparently lifeless state. Within half an hour of the accident he was seen by a surgeon, who bled him to ℥xx. which relieved him. He was then placed on a shutter on his back, and brought to the hospital. On his arrival he was seen by Mr. Worry, one of the house-surgeons, at which time the accident had occurred two hours. He was carefully examined, and it was found that he had received an extensive fracture of the walls of the chest. The three or four upper ribs on the left side were satisfactorily ascertained to be broken between their angles and the sternum, and one or two of them in more than one place. The middle bone of the sternum was fractured, and slightly beaten inwards. The clavicle was depressed, but not fractured. There was a slight degree of emphysema on the left side. His breathing was very difficult and laborious; his countenance pale; pulse small and 110. Upon placing the hand on the chest, the crepitus communicated, not only to the touch, but to the ear, was such as made all present who examined him, shudder. His whole appearance resembled that of a man just sinking from effusion of blood into the chest. He was, however, bled to ℥xx. which produced syncope. A wide rib-roller was placed tightly round the chest, and his head and shoulders elevated by means of pillows placed behind his back. In this state he was left; no one expecting him to survive the night.

21*st*. 8 *o'clock, a. m.*—To the surprise of all who had seen him the preceding night he was found still alive; having suffered much difficulty of breathing throughout the night; his pulse is 100, and stronger. The emphysema has not perceptibly increased; ordered,

V. S. ad ℥xx. Hydr. submuriatis, gr. vi. jalapæ, gr. v. quarta quaque hora, cum misturæ ammoniæ, acet. ℥iss. et liq. antimonii tart. ♏ xx.

12 *o'clock, Meridie.*—He was seen by Mr. Earle, who directed the figure of eight bandage, as usually employed for fractured clavicle, to be applied over the chest, on which was placed a large piece of wetted pasteboard; as the rib bandage could not be brought so high as effectually to confine the fractured surfaces.

9 *o'clock, p. m.*—The bowels have been freely evacuated; and he is considerably relieved. Pulse strong, full, and 108; hard dry cough.

V. S. ad ℥xxx. which produced syncope and a considerable moisture on the skin.

22*nd*. 8 *o'clock, a. m.*—Has had some sleep during the early part of the night, but towards the morning the cough became very troublesome. Tongue moist, and but little coated. The bowels being freely evacuated, the calomel and jalap was omitted.

10 *o'clock, p. m.*—Heat of skin increased; pulse 96 and again strong; cough troublesome, and attended with considerable wheezing.

V. S. ad ℥xvj. which produced syncope.—Emplastrum lyttæ sterno.

23*rd*. 8 *o'clock, a. m.*—Considerably better; has had three or four hours sleep during the night; the cough has not been so troublesome, nor is the wheezing so great; pulse 100 and much softer.

10 *o'clock, p. m.*—The pulse has increased in hardness, and the skin become hot and dry.

V. S. ad ℥xv. which produced syncope. The blister to be dressed with the savine cerate. He has taken nothing except tea and arrow-root since his admission into the hospital.

24*th*. 8 *o'clock, a. m.*—In every respect better. Has had some comfortable sleep at intervals during the night. The cough less urgent, and attended with an expectoration. The blood drawn the last morning had assumed the inflammatory buffy appearance which had not been the case at the previous bleedings. Pulse soft, and 86; skin moist.

R. Misturæ ammoniæ acet. ℥iss. liquoris antimonii tart. ♏x. vini semin. colchici ♏xv. tertia quaque hora sumend.

25*th*. The patient has been going on favourably, with the exception that expectoration has become very copious and of a purulent nature. The countenance, and conjunctivæ of the eyes have taken on the jaundiced appearance; the bowels *purged*, and the stools of a light colour.

Discontinue the mixture ordered yesterday. Pulv. ipecacuanhæ comp. gr. v. cum misturæ cretæ, ℥iss. sextis horis vel post sing. dejectiones liquidas.

26*th*. Much better; the bowels quiet.

27*th*. The expectoration still considerable, and of a purulent nature; pulse soft, and 88; tongue moist. Discontinue the chalk mixture, and Dover's powder. Ordered, misturæ camphoræ, ℥iss. tincturæ camphoræ comp. ʒj, pulveris myrrhæ, gr. x. ter in die.

28*th*. The jaundiced appearance still considerable. Ordered pilulæ hydrargyri, gr. iij. extracti papaveris albi, gr. v. omni nocte.

Contr. mistura cum camphora et myrrha.

29*th*, 30*th*, and 31*st*. Has continued to improve. Expectoration not so great; and there is a mixture of mucus with flakes of blood.

*April* 3*rd*.—Going on well. There is every prospect of his doing well. The blister is kept open. He takes rice milk, and a little strong broth.

Pergat. mistura cum camphora et myrrha.

10*th*. His health is improving, and he is gradually gaining strength. The expectoration has ceased.

23*rd*. Discharged cured.

*Case* 2. John Church, æt. 44, was admitted into Rahere's ward, on the 3d of June, under the care of Mr. Lawrence.

This patient, who is a porter, of temperate habits, fell with violence against the edge of some pavement, and thus received an injury to his side, which, upon examination, was found to consist of fracture of two or three of the middle ribs, near their angles, accompanied with an emphysema of the adjacent integuments, about a hand's breadth in extent. He complains of acute pain when any attempt is made to cough, or enlarge the chest by inspiration; pulse natural. Previous to admission he was bled to ℥xx. A broad bandage was now applied tightly around the chest, thus rendering it immovable.

Hydr. submuriatis, gr. iv. Jalapæ, gr. x. Mist. sennæ comp. ℥iss. post horas duas. (Milk diet.)

7 *o'Clock, p.m.* The pulse has become hard, and 98, and the breathing hurried; bowels freely opened.

V.S. ad ℥xxx. Misturæ ammoniæ acet. cum liquoris antimonii tart. ʒss. sexta quaque hora.

4*th.* Immediate relief was obtained from the bleeding. Has slept well, and is perfectly easy this morning; no further diffusion of air perceptible.

Cont. mist. ut heri prescripta.

5*th.* Has gone on in every respect favourably. There has been no extension of the emphysema; no pain in the side, nor feverish symptoms.

Pergat. Mist. ammon. acet. cum liq. antimonii tart. et mistura sennæ c. pro re nata.

20*th.* No remains of the emphysema can be discovered. The bandage is still worn, and he is allowed to walk about the ward.

Int. mistura ammon. acet. cum liq. antim. tart.

24*th.* Discharged cured.

*Case* 3. George Freeman, æt. 26, a brewer's waggoner, and a stout muscular man, was admitted into Rahere's back ward, on the 14th of June, under the care of Mr. Vincent.

He states that, when lowering a cask from his waggon into a cellar, by means of a rope, turned round a post, the post gave way, and the cask threw him with violence against the edge of the door-frame. On examination, fractures of two or three of the ribs, just below and a little anterior to the inferior angle of the scapula on the left side, were discovered, together with an emphysema of the adjacent integuments, in extent about equal to an octavo leaf. His breathing was not perceptibly affected, nor did he complain of pain or uneasiness, except upon motion, or an attempt to cough. A broad belt was tightly buckled around the ribs, to confine their motion.

V.S. ad ℥xx. hydrargyri submuriatis, gr. iij. Jalapæ, gr. xij. statim sumend. et mist. sennæ, c. post horas duas. (Milk diet.)

15*th.* Has passed a restless night; pulse hard, and increased in frequency; breathing hurried; no perceptible increase of the emphysema; the bowels have been well opened.

V.S. ad ℥xxx. Misturæ ammoniæ acetatis, ℥iss. cum liquoris antimonii tart. ʒss. sexta quaque hora.

16*th.* The bleeding yesterday was followed, in a quarter of an hour, by complete relief to the breathing; the blood presented no inflammatory characters. He has slept well during the night, and is quite easy this morning. The frequency of the pulse is diminished; the bowels are well opened.

Cont. mistura salin. ut antea.

*July 2d.* He has kept perfectly quiet. The belt round the ribs has been retained, and a dose of the mist. sennæ c. occasionally administered, to keep the bowels open. He has gone on in every respect favourably, and the whole of the air has been absorbed from the interstices of the cellular texture.

Int. mistura salin.

4*th.* Discharged cured.

*Remarks.* The three preceding cases illustrate the beneficial mode of treatment first recommended and practised by Sir William Blizard. They tend to show the necessity for, and advantages resulting from, the copious depletion which that gentleman inculcated. Considering, in cases of emphysema, the probability that air has been effused into the cavity of the chest, between the pleura costalis and pulmonalis, occasioning a collapse of the corresponding lung; consequently, the additional labour which the sound one will have to undergo, as also the evil consequences likely to arise from a congestion in that organ, at a period when an additional energy is required; the indication which immediately suggests itself is, to get rid, as quickly as possible, of that quantity of circulating blood, which, by its continuance in the system, would not only prove a burthen to the sound lung, but favour the disposition to pleuritis. Upon this principle, copious venesection was employed, and to it I refer the relief which followed in each case.

Mr. Abernethy, in the second volume of his "Surgical Works," has shown it to be an error which those surgeons entertain, who consider the employment of a belt or bandage at the commencement of these cases, more likely to occasion the insinuation of air between the two pleuræ; and that, by rendering the walls of the chest immoveable, it not only prevents that pain being produced, which the enlargement of the chest by the intercostal muscles would necessarily occasion, but, by its pressure, prevents the further escape of air through the wounded lung, and thereby facilitates the healing of the wound.

Whether, in the generality of these cases, the difficulty and oppression of the breathing arise from an impediment presented to the sound lung in the performance of its functions, by the pressure from the mediastinum, or by the additional quantity of blood transmitted to it by the pulmonary artery, or by both, I am unable to determine. Certain, however, it is, that the copious abstraction of blood sometimes altogether removes, and seldom fails to alleviate the most urgent symptoms.

---

The two following cases strikingly illustrate the happy effects which result from a judicious position of the limb, in transverse fracture of the patella.

*Case* 1. William Denton, æt. 27, was admitted into Kenton's ward on the 1st of April, under the care of Mr. Lawrence, having, in an attempt to save a fall, whilst carrying a load upon his shoulder, fractured his right patella transversely. He immediately lost all power of extending the limb, and supporting the trunk of the body on that side. The upper portion of the bone was drawn up by the action of the extensor muscles, and a space of two inches intervened between the broken surfaces of the bone. The fore part of the knee-joint was swollen, from ecchymosis, and of a livid colour; but not attended by much pain.

He was placed upon his back in bed; the trunk of the body slightly elevated, to relax the rectus muscle, and the heel raised, by means of pillows, four inches above the anterior and superior spinous process of the corresponding ilium.

Hydr. submur. gr. iv. Jalapæ, gr. x. statim. (Milk diet.)

Wet rags to be constantly applied to the joint.

2*d*. The knee is painful, and much swollen; bowels freely opened.

Hirudines xx. genu.

Nothing of interest occurred in the further progress of the case. The bowels were kept open by means of calomel and jalap, and house-physic; (mist. sennæ c.) the temperature of the joint regulated by means of cold washes; a state of the most perfect quietude enjoined; and the former position of the limb accurately observed until May 11th, when a little gentle motion was allowed, and daily increased until the 18th, (about six weeks from the occurrence of the accident) when he was discharged, cured.

*Case* 2. William Jones, æt. 40, was admitted into Rahere's ward, May 12th, under the care of Mr. Lawrence, with a transverse fracture of the right patella, occasioned by falling upon his knee on the edge of a curb-stone, when jumping from a cart. Great swelling of the joint followed; the power of extension of the leg was immediately lost, and any effort made to support the trunk of the body on that side proved ineffectual. The upper portion of the bone was drawn upwards by the action of the extensor muscles, while the lower retained its natural situation. An interval of two inches existed between the fractured ends.

He was placed upon his back in bed, with the trunk and extremity exactly in the same position as in the former patient, and ordered—

Hydr. submur. gr. iv. Jalapæ, gr. x. statim. Mixturæ sennæ, c. ℥iss. post horas duas. (Milk diet.) Wet cloths to the joint.

13*th*. The knee is painful and very much swollen; skin hot and dry; pulse hard and 96; bowels not relieved.

V. S ad ℥xx. hirudines xxx. genu, misturæ sennæ c. ℥iss. statim. et repr. 2ndâ quâque horâ donec alvus plenè respond.

14*th*. Bowels have been freely acted upon; pulse 90 and full; skin hot; tongue white and dry; knee swollen, tense, and painful.

V. S. ad ℥xvj. misturæ ammoniæ acet. cum liquoris ant. tart. ʒss. sexta quaque hora

15*th*. Pulse 82 and soft; skin and tongue moist; knee less painful (pergat.)

20th. The joint is free from pain and the swelling rapidly diminishing; the pulse natural; tongue clean; bowels regular. Intr. mistura salin. ac liq. ant. tart. pt. mistur. sennæ comp. pro re nata.

The limb was carefully kept in the position in which it was first placed, and the patient directed to keep it in a state of perfect quietude until the 13th of June; when a little gentle motion was allowed, and daily increased until the 18th, when he was discharged, cured.

*Remarks.* It will appear that these cases were treated simply by relaxing the extensor muscles of the leg; neither bandages, splints, nor straps, being employed in the progress of either case. Upon a careful examination of the patella, in each patient, on the day of discharge, so closely were the divided surfaces found to be approximated, and so great was the power which each individual possessed in the extension of his leg, and support of the trunk of his body on the affected limb; that, had a person unacquainted with the usual mode of reparation in these cases, examined the bone, he would, I think, have unhesitatingly declared that an osseous union had taken place.

But, bearing in mind the three circumstances, first, that patients are not always so conformable to admonition as these have been; second, the unavoidable movements which must sometimes necessarily take place from spasm and various other causes; and third, how advantageous it is to the patient, at all times, to obtain a short ligamentous union; both as fortifying him, in great measure, against that weakened power of extension in the affected limb, and that liability to fracture of the patella in the opposite one which inevitably attends an opposite state of it: it will appear prudent, and advisable, in order to ensure more certain success, either to adopt, in conjunction with the foregoing position of the limb, the mode of treatment recommended by Sir Astley Cooper in his work on "Dislocations and Fractures of the Joints," that of buckling a leathern strap around the thigh above the broken and elevated portion of bone, which is to be secured by another continued from it underneath the foot; or, to confine the upper as nearly as possible in contact with the lower portion by means of a bandage passed alternately round the lower part of the thigh and upper part of the leg in the form of a figure of eight. Of course neither straps, nor bandages will be employed, until the inflammation following the accident, has subsided.

---

The following case illustrates the result which may generally be expected to take place subsequent to the operation of femoral hernia, after so protracted a period of strangulation.

Mary Roberts, æt. 67; who is of a delicate habit, is married, and has borne children, was conveyed to the hospital on the 28th of June; and states, that for forty years she has been the subject of rupture; the reduction of which, previous to the present time, she has readily effected.

On Monday last, (25th) after having walked some distance, the gut descended; but she was not conscious of having used any great effort or unusual exertion. She resorted to her accustomed mode of reduction (the recumbent posture; flexure of the thigh, and warm fomentations) but without success. She then applied to a medical man, who had frequently, from the period of her application to him to the time of her admission into the hospital, made use of the taxis, but with the same unfavourable result.

*June 28th*, half-past three o'clock P. M. She presented the following symptoms: A feeling of tightness across the epigastric region; nausea, vomiting, and the rejection of fæcal matter; pulse 100, small, and hard; great anxiety and restlessness; cold extremities; no stool since Monday. A well defined and moveable tumour, about the size of a walnut, with its longest diameter in the transverse direction, was observed on the upper and inner part of the right thigh. From the neck of the sac, extending from the femoral ring, through the upper part of the large oval depression formed by the two portions of the fascia lata immediately below the crural arch, the body of the hernia might be traced turning over the semi-lunar edge or falciform process of the fascia, and insinuating itself into the loose cellular tissue at the bend of the thigh, in a direction upwards and outwards; thus forming a right angle with the neck; whilst its fundus rested upon the front surface of Poupart's ligament, where it moved freely. The part was tense; the neck of the sac acutely painful; and the tenderness had extended from thence over the whole body and fundus of the tumour, and adjacent part of the abdomen.

The thigh on the affected side was bent to a right angle with the body, rotated inwards, and carried across the opposite thigh; thus relaxing, as much as possible, the fascia concerned in the formation of the stricture:—The taxis was then attempted, and the hernia endeavoured to be moved in a direction downwards and inwards, then upwards; but without success. Considering the urgency of the symptoms, the time which had elapsed since the period of strangulation (72 hours) and, consequently, the inefficacy and mischief likely to attend the further employment of the taxis under any circumstances of depletion or collapse; it was judged expedient to send immediately for Mr. Earle, as the only chance the patient had, rested in the uncertainty of an operation; as it unquestionably was a great uncertainty, so small is the opening through which the intestine passes, and so tightly must it consequently have been girt for a space of three days.

Mr. E. arrived at half-past seven, and did not hesitate immediately to recommend and perform the *operation*.

The patient being seated upon a table, in a favourable light, with her head and shoulders slightly elevated by means of a pillow, the hair was removed from the surface of the swelling, and an incision commenced about an inch above the crural arch, which was continued over the front of the tumour in a perpendicular direction to the same extent below; dividing 1st, the integuments; 2nd, the condensed adipose and cellular tissue constituting the fascia superficialis; 3rd, the fascia propria, which tunic had much the appearance of thickened and diseased omentum. Turning this to either side, the peritoneal sac was exposed, which was thickened, (as in fact were all the coverings with the exception of the integuments); a portion of it was taken hold of by a pair of forceps, and a small aperture made into its lower part, through which a director was introduced, and its front surface laid open by means of a probe-pointed bistoury. No fluid escaped. Another covering was then perceived which had given way, at one small point, by the pressure of the finger nail in separating it from the peritoneal covering; and from it escaped an inodorous bloody serum. At first sight, this was thought to be the bowel; but no circular arrangement of the vessels being discovered, a director was introduced into its interior, when it most readily gave way, and was found to be an adventitious membrane which adhered very firmly to the bowel underneath. The adhesions were very carefully divided by means of an eyed probe, and a small knuckle of the bowel of a reddish brown or chocolate colour was exposed. The probe was then carefully introduced between the internal surface of the bowel and the edge of Gimbernat's ligament, thus making way for the director, which was introduced with its groove directly opposite the ligament, and a few fibres of the latter divided by means of Sir Astley Cooper's hernia knife, which has about half an inch of cutting edge in its middle. The bowel was then very readily returned into the abdomen, and the finger passed after it, discovered its œsophageal extremity very much increased in calibre; being rammed with hard fæces. Two sutures were employed to bring the edges of the wound in apposition, and two wetted compresses placed over it, which were secured by a roller passed round the pelvis and thigh in the form of a figure of eight. She was immediately laid in bed, and the following injection administered.

Misturæ sennæ c. ℥viij. aquæ tepidæ ℥ij. ft. enema.

No relief followed. After two hours the following was directed to be thrown up the rectum, and to be repeated every two hours, if necessary.

Ol. ricini. mist. sennæ c. aa ℥iv. ft.

enema. ½ *past* 9 *o'clock.* No motion; pulse small, hard and 108; great tenderness in the right groin and abdomen; skin hot and dry.

V. S. ad ℥xij. Hirudines xxx. parti affect. 12 *o'clock.* The injections return but without any admixture of fæcal matter.

Magnesiæ sulphatis, ʒj. aquæ menthæ tivæ, ℥iss. 2ndo quaque hora.

*June* 29*th,* 8 *o'clock.* A. M. Has had two slight motions, but apparently from the large intestines only; pulse small and 160.

Ol. ricini, ℥j. 2da quaque hora.

*Ten o'clock,* P. M. No evacuation from the bowels; is delirious; great pain and tenderness in the abdomen; pulse rapid and intermitting.

Olei terebinth. ʒij. mucilaginis acaciæ, ʒiij. aquæ menthæ pip. ℥j. ft. haustus statim sumendus. Pultis, ℥viij. olei terebinth. ʒij. ft. enema statim injiciendum.

30*th.* The means employed were followed by no relief and she expired at five o'clock this morning.

*Sectio Cadaveris, eight hours after death.* The peritoneum, covering the intestines and lining the abdominal parietes bore evident marks of inflammation; it was highly vascular. The intestine which had been strictured was found to be a portion of the ileum, and was thickened by the effusion of lymph between its coats. The mark of the stricture was very apparent, extending round three fourths of the circumference of the gut. Ecchymosis had taken place in this situation to a small extent between the peritoneal and muscular coats of the bowel. The mucous membrane was rather unusually vascular; but not ulcerated. The feculent matter contained in the ileum was soft and rather copious. The cæcum, colon, and rectum were distended by the injections. A few fibres of Gimbernat's ligament only were divided; and Mr. Earle was quite satisfied that this alone caused the strangulation. No morbid appearances were observed in any other viscus.

---

The two following cases exemplify the efficacy of a local sedative in the treatment of the primary phagedænic ulcer.

*Case* 1. John Macnamara, æt. 24; of temperate habits, was admitted into Lazarus' back ward the 22nd of June, under the care of Mr. Earle.

Three weeks ago, and about three months after connexion, he perceived a slight excoriation immediately behind the corona glandis, which, not being painful, he was induced to disregard, and pursued his daily occupations. A sore was produced in the situation of the excoriation, which gradually enlarged and became painful. He has made use of no other remedy than local ablution.

*June* 22*nd.* An excavated sore, about the size of a shilling, with a ragged and hard edge, and a foul, ash-coloured, and irregular surface discharging a thin sanies, together with a slight degree of redness in the surrounding integuments, occupies the left side of the basis of the glans, and a corresponding portion of the adjacent prepuce. The glands in the groin are not affected. The local pain is severe, particularly at night. Pulse 80, and full—skin hot and dry—tongue white.

V. S. ad ℥xij. hydrargyri submuriatis, Pulveris Jacobi ana gr. v. hæc nocte. Misturæ sennæ c. ℥iss. cras mane. Lotio opii (opii drachmam cum semisse, aquæ octarium) parti affectæ. (milk diet.)

23*rd.* Sore presents much the same appearance, but is free from pain—pulse reduced to 70.

Rep. hydr. submur. et pulvis Jacobi hæc nocte. cont. lotio opii.

25*th.* There is a slight inflammation in the right eye.

Hirudines viij. tempori dextro.

26*th.* Iritis threatens. The pupil is contracted—the eye painful when exposed to light—the conjunctiva reddened, and a faint pink-coloured zone appears around the cornea. The sore on the penis is destitute of pain and has assumed a more healthy appearance. The bowels are freely open.

Appl. c.c. tempori dextro, et detr. sanguis ad ℥xvj. Extr. belladonnæ supercilio. antimonii tartarizati gr. j. hæc nocte. Cont. lotio opii. cum acidi nitrisci ♏ iij. ad ℥j.

28*th.* The symptoms of inflammation in the eye have subsided; the foul appearance of the sore has disappeared, and is succeeded by a healthy granulating surface. His general health is good.

Cont. lotio; et mistura sennæ c. pro re nata.

*July 3rd.* The sore is cicatrising rapidly, and not more than half its original size. Pergat.

*12th.* The sore is healed, and he was discharged cured.

*Case 2.* John Corbalt, æt. 30; accustomed to live freely, observed a small excoriation on the outer part of the prepuce about a fortnight ago, and a week after connexion, which gradually enlarged and became very painful. He applied at the hospital, and was admitted into Lazarus' back ward, under the care of Mr. Earle.

*June 29th.* An irregular and excavated ulcer, an inch and a half by ¾ in extent, with a hard and elevated edge, a dark brown, foul, and irregular surface discharging an offensive, thin and bloody matter, is situated on the fore and outer surface of the prepuce. He complains that it is painful, more especially at night. His constitution is not affected.

Hydrargyri submuriatis, gr. iv. pulveris antimonialis, gr. v. hora somni sumend: (milk diet) let a piece of lint dipped in opium lotion, and afterwards a cataplasm made with the opium lotion, be applied to the surface of the sore.

*July 2nd.* The foul appearance of the sore is removed, and its surface covered with healthy granulations. The pain arising from it has ceased. The following lotion was directed to be applied to the part night and morning.

Lotionis opii, ℥j. acidi nitr. ♏ iij. M.

*8th.* The sore is cicatrising. Cont. lotio.

*16th.* The ulcer has completely healed, and he was discharged, cured.

*Remarks.* Mr. Earle considered the two former cases, well-marked instances of the primary phagedænic ulcer; and, as the constitutional disturbance of the former patient was only that which it was thought a general bleeding would remove, and the latter altogether free from it, he conceived that by allaying the irritability of the part by means of a local sedative, the ulcers would assume a healthy character. The favourable progress of the cases has confirmed the opinion Mr. E. entertained.

The following case shows the efficacy of the local application of the concentrated nitric acid; and the mode of treatment sometimes adopted by the surgeons of this hospital in cases of primary phagedænic ulceration.

Anne Wheeler, æt. 45; was admitted into Patience ward the 29th of June, under the care of Mr. Earle.

She was free from disease, when, seven weeks ago, she perceived a small pimple on the inner edge of the left labium, which itched intolerably, broke, became a sore, and spread rapidly; so that in the course of a fortnight, (during which time she drank freely of spirituous liquors and porter,) it had acquired its present size. From that time to the present, (a period of five weeks,) it remained stationary, but gave her very acute pain. She employed no other remedy than simple ablution.

*June 29th.* An excavated sore, half an inch in depth; with a spongy, foul, and dusky-coloured surface, secreting an offensive bloody sanies; has extended about an inch within the left side of the vagina, and destroyed the left nympha and lower half of the left labium, including a circular portion of the soft parts, about the size of a crown piece, below, and to the left of the vulva. She describes the pain she suffers as excruciating. Her general health is not, however, affected. The surface of the sore to be dried with lint; afterwards freely soaked with the undiluted nitric acid, by means of a piece of lint wrapped round a probe; and then covered with dry lint. To take, immediately after the application, tincturæ opii ♏ xxx.

*30th.* The application of the acid caused very great pain for about 20 minutes, when it entirely ceased, and has not since recurred. It has produced a slough over the whole extent of the ulceration.

Hydrargyri submur. gr. iij. jalapæ gr. x. statim. pulveris ipecacuanhæ c. gr. x. bis in die.

*July 2nd.* The slough has separated, and a healthy surface remains. She has remained quite free from pain.

Cont. pulvis ipecacuanhæ c. et capiat mistur. sennæ comp. ℥iss. pro re nata.

*5th.* The ulcer has a perfectly clean surface. There is not the slightest pain attending it.

Cont. medicamenta.

*6th.* A small unhealthy spot exists at the superior edge of the sore. Let the application of the acid be repeated, and followed by the administration of thirty drops of laudanum.

*7th.* The application again gave great pain, which ceased in a quarter of an hour. A small slough is produced in the situation of the foul spot.

Cont. pulvis ipecac. c. et mist. sennæ, c.

*9th.* The slough has separated, and the whole surface of the sore presents a clean aspect. (Pergat. lotionem opii. Intr. pulvis ipecacuanhæ c.)

*16th.* The ulcer is diminishing in size. Her health continues good. The lotion is discontinued, and the sore dressed with mild cerate. Cont. mist. sennæ, c. p. r. nata.

*August 7th.* The sore has completely cicatrised; her health is good, and she is discharged.

The following is an instance of phagedænic ulceration, successfully treated by the proto-nitrate of mercury, after the failure of nitric acid.

Abraham Perkins, æt. 19; was admitted on the 7th of June, under the care of Mr. Earle; and gives the following history of his complaint.

Four months ago, and a week after connexion, he contracted an indurated chancre on the prepuce, close to the frænum, which was succeeded by a bubo in the groin, and for which he was admitted into the hospital, under the care of Mr. Lawrence. The sore healed completely in a fortnight, but his mouth was not affected; and the bubo was not quite healed, when he was made an out-patient. It completely closed, however, in a week afterwards. He declares most positively that he had not subjected himself to fresh contagion, when, three weeks ago, he perceived a small white pimple in the situation of the original sore, which broke and bled a little. During the four next days it gradually, but slowly increased, and then remained stationary for about a fortnight, at which time, after drinking freely, it became excessively painful, and spread rapidly up to the period of his admission.

*June 7th.* The whole of the under surface of the glans and a corresponding portion of the prepuce, including the frænum, are occupied by an excavated sore, the edges of which are hard and well defined; the ulcer presents an irregular, bloody, foul, and dark-coloured surface, and secretes at its edges an offensive sanies. The surrounding parts are painful and tumefied; he is feverish, tongue white, skin hot and dry, pulse quick.

V. S. ad ℥xvj. hydrargyri submur: gr. iij; jalapæ, gr. xij. statim. Misturæ salin: 4ta. quaque hora. Pulv. ipecacuanhæ c. gr. xv. hora somni.

The surface of the sore to be dried with lint; the surrounding parts to be smeared with lard; the sore to be freely soaked with the undiluted nitric acid; dry lint to be applied for a quarter of hour, and afterwards a bread and water poultice. (Tincturæ opii, ♏ xxx.) Milk diet.

*9th.* The application of the acid gave great pain, and produced a slough, but has not produced any improvement in the appearance of the sore, or permanent remission of the pain. To have the acid freely re-applied and to take immediately afterwards tincturæ opii, ♏ xxx. olei ricini, ℥i. statim. Tincturæ opii, ♏ xv. misturæ camphoræ, ℥iss. hac nocte. Cont. mistura ammonia acetatis.

*11th.* The application of the acid has been equally inefficacious. The ulceration has enlarged, and the pain is very severe.

Pulv. ipecacuanhæ comp. gr. xv. hora somni omni nocte.

*12th.* He is in much pain; has headache, a white tongue, hot and dry skin, and quick pulse.

V. S. ad ℥xvj. Olei ricini, ℥j. statim. antimonii tart. gr. j. aquæ menthæ pip. ℥iss. post horas duas et repet. si opus sit. Let the following application be used in the same manner as the nitric acid; the sore afterwards covered with dry lint and forty drops of laudanum administered.

Hydrargyri nitratis, ʒss. acidi nitrici, ℥j. ♏.

*13th.* The application produced great pain, which was followed, in twenty minutes, by perfect ease. A slough is formed over the whole surface of the sore.

*16th.* Has remained perfectly free from pain; the surrounding redness and tumefaction are subsiding, and the slough has separated. He feels weak and low.

Extracti sarsæ, ℥ss. bis die. Cont. pulvis ipecac. c. et mistura salin.

*26th.* The sore has assumed a perfectly healthy character; the excavation is filling with healthy granulations, and the sore contracting. He is altogether free from pain. Let the sore be dressed with the balsam of Peru upon lint. Cont. medicamenta.

*30th.* The sore is healing rapidly. (Meat diet.)

*July 12th.* The ulceration has completely cicatrised, and he is this day discharged.

*Remarks. Sloughing phagedæna,* as observed in St. Bartholomew's Hospital, seems to be a local affection, produced by inattention to cleanliness, by cold and damp, improper food, and the too frequent use of spirituous liquors. Such causes seem to aggravate so much the consequences of the application of morbid secretions, that a peculiar train of symptoms is produced, which, in their turn, affect the constitution.

When, however, the local affection is destroyed, the constitutional effects quickly disappear. This seems to be effectually done by the nitric acid, which, by penetrating deeper than the other cauteries, more completely destroys the morbid part. After its application the part usually assumes the appearance of a healthy sore; but, if the acid has not been sufficiently strong, or if the ulcer has not been sufficiently soaked with the application, the morbid part will not be completely destroyed; either the whole or a portion of the sore will continue to present the usual unhealthy appearance, and will require another application for its destruction.

When *phagedænic sores* have taken on an unfavourable appearance, it will often be of use to apply the concentrated acid. In these cases, however, the same success will not be obtained as when applied to the sloughing species: in some cases it seems to aggravate the disease, much as in the above case. In these cases the greatest advantage is obtained by attention to the general health, and the local application of an oxyde of mercury, as the black or yellow wash. In other cases of this kind the stimulus of a solution of the unchemical preparation called above, proto-nitrate, is of service. Dr. T. A. Wise informs me that he has seen this last preparation employed with the greatest success at the Hospital of St. Louis, in Paris, by that excellent surgeon, J. Cloquet, in cases of phagedænic ulcers of the throat or nose (Lupus), &c. In these cases, by means of a dossil of lint, the ulcer and surrounding redness are washed over with the solution. It gives considerable pain at the time, which goes off in about half an hour. In a day or two a small slough separates, leaving the sore in a healthy state. The same gentleman informs me that he has frequently seen the most severe and extensive ulcers of the throat and nose completely cured after the second or third application of this preparation.

In conjunction with these means, internal anodyne remedies, such as pulvis ipecacuanhæ comp., conium, &c. are employed in this hospital, and generally with great advantage. I have not seen mercury employed for the primary forms of the phagedænic disease; but in those cases where it has been administered to patients previous to admission into the hospital, a most decided aggravation of the symptoms has been produced. In the secondary ulceration it is used, however, either internally in the form of oxymuriate with decoction of sarsaparilla, or externally in the cinnabar fumigation, or yellow wash (hydr. oxymur. gr. j, liquoris calcis, ℥j.), and with manifest advantage.

The secondary phagedænic symptoms are not confined, as Mr. Carmichael has observed, to the primary phagedænic ulcer. A case is at present in the hospital where they have followed gonorrhœa, and several others have been admitted during the past winter consequent on the same stock of primary infection.

9 √

---

ULCERATION OF THE LARYNX, WITH DIFFICULTY OF BREATHING AND SWALLOWING, AND GREAT HOARSENESS.

William Cox, æt. 32; a spare unhealthy looking man, was admitted into Kenton's ward on the 26th of April, under the care of Mr. Lawrence.

The patient, whilst confined in prison about a year ago, was attacked with a hoarseness, which has continued ever since. For the first nine or ten months it was not attended with pain or difficulty of either respiration or deglutition. About nine weeks ago he felt, for the first time, a pain in the larynx; and about the same time deglutition and respiration became difficult, and have continued gradually to increase. During the last three weeks deglutition has been so painful as almost to preclude the possibility of his taking a necessary supply of food. He has been almost free from cough until the last 12 or 14 days. He has taken no medicines for his complaints.

At present he is enabled to articulate only in a whisper; respiration is difficult and attended with a wheezing noise; he has a cough, attended with a copious expectoration of thick, viscid, but not purulent matter, which is particularly distressing to him when in the recumbent position: deglutition is so painful as to prevent his taking any solid food: there is a constant dull pain in the larynx.

Hydrargyri submur. gr. ij. opii, gr. ½, ter in die. Misturæ sennæ c. ℥iss. pro re nata.

30*th*. No perceptible alteration. Hirudines xij. gutturi et postea emplastrum lyttæ.

*May* 4*th*. His mouth is affected by the calomel. Respiration and deglutition less painful and difficult; hoarseness much the same.

Intr. cal. et opii. Misturæ ammoniæ acet. ℥iss. sexta quaque hora.

15*th*. Has continued gradually to improve. Pergat.

18*th*. The symptoms have become worse: he complains of great pain in the larynx.

Emplastrum lyttæ gutturi.

21*st*. Continues to get worse: the cough is very distressing at night, and he complains of pain in the chest; takes scarcely any food, and is greatly emaciated.

Hirudines xij. gutturi.—Emplastrum lyttæ amplum sterno.

25*th*. Deglutition less difficult; in other respects nearly the same.

30*th*. For two or three days he has been able to take the necessary quantity of food, with very little pain or difficulty. Last night deglutition again became very difficult, and his cough very distressing.

Linctus cum scilla.

*June* 1*st*. The linctus affords no relief. Let it be discontinued, and a table-spoonful of the following mixture taken occasionally.

Tincturæ camphoræ comp. ℥j. misturæ amygdal. ℥vij. ♏.

6*th*. Much benefited by the mixture.

9*th*. He has again suffered very much from difficulty of respiration for the last two days. Yesterday he was attacked with spitting of blood, which continued during the night: the quantity however was small, and has now entirely ceased.

11*th*. Is much exhausted; pulse small and weak.

Vini, ℥vj. quotidie.

15*th*. For two days after the last date he was better; but has been getting since then gradually worse. Respiration and deglutition are now in the highest degree difficult and painful, and he is evidently sinking.

16*th*. Died at six o'clock this morning.

Upon the *sectio cadaveris*, the following state of the parts was found:

Extensive superficial ulceration of the mucous membrane: it occupied one-half of the anterior, and the whole of the posterior surface of the epiglottis, the cavity of the larynx generally, and about an inch of the upper end of the trachea. The neighbouring portion of the membrane was thickened, and rather indurated: that lining the trachea and bronchi very vascular, slightly swelled, and covered by a yellow secretion. Although the mucous membrane covering the ligaments of the glottis was thickened, the opening still appeared large enough for respiration.

Very firm and general adhesions of the pleuræ; both lungs filled with small tubercles, many of them ulcerated; the intermediate spaces healthy: a few small depositions of a chalky substance in both lungs.

The pharynx and œsophagus healthy.

The contents of the abdomen healthy.

The arachnoid membrane of the brain thickened and slightly opaque: great fulness of the vessels, with effusion of serum into the cellular texture of the pia-mater: great fulness of the vessels in the substance of the brain.

Jane Mudie, æt. 26; and female child, æt. 16 months; were admitted into Magdalen's ward on the 31st of March, discharged, and re-admitted on the 21st of May, under the care of Mr.

Lawrence; the mother having gonorrhœa, subsequently elevated sores on the labia, perineum, and nates, afterwards ulcers of the tongue and lip; the child, purulent ophthalmia, pustular eruption, vaginal discharge, excoriation with elevated ulcerations of the anus and perineum: relapse of ulceration, and iritis of the left eye.

*March 31st.* The mother observed a discharge from the vagina, with scalding in making water about four months before the birth of this child; the former continued until three months ago, when she noticed large elevated sores on the inner side of the labia, others on the perineum and around the anus, which are now elevated, warty, and ulcerated excrescences. She does not seem to have had any other venereal affection.

The infant when born was large and healthy, but in three days afterwards was attacked with purulent ophthalmy in both eyes, for which it was treated at the Ophthalmic Infirmary, Moorfields, and soon got well. Five months after birth a pustular eruption appeared on its neck, which soon disappeared; a discharge was then observed from the vagina, the labia swelled, and were extremely excoriated; these symptoms subsiding, a number of elevated, flat, warty excrescences appeared on the perineum, and around the anus, which now present excoriated heads, like those of the mother.

Ordered for the mother, pilulæ hydrargyri, gr. v. nocte maneque—lotio nigra partibus exulceratis.

For the child, hydrargyri cum creta, gr. iv. bis in die.

The symptoms soon yielded to this treatment, both in the mother and infant, and they were discharged, apparently cured, on the 18th of April.

Re-admitted on the 21st of May.

The mother has an ulcerated fissure on the middle of the apex of the tongue, with swelling, redness, and excoriation of the entire apex, and considerable pain in moving the part; she has also a whitish superficial sore, as large as a sixpence, on the mucous membrane of the lower lip. These ulcerations have existed for a fortnight: there has been no return of ulceration on the perineum or organs of generation.

The child has considerable excoriation and ulceration round the anus, which reappeared in a week after her discharge from the hospital. She has also iritis of the left eye in a mild form, which the mother ascribes to a cold from having the head wetted. The iris is darker than the sound eye, and has lost its brilliancy, the pupil a little contracted, slight pink redness in the sclerotic coat around the cornea, slight intolerance of light. The symptoms of iritis commenced three days ago.

For the mother, hydrargyri oxymuriatis, gr. ⅛ ex decoct. sarsæ, ℥j. quotidie.

For the child, hydr. cum creta, gr. v. mane et vespere quotidie.

*24th.* A small red and painful spot is observable on the side of the tongue, near its basis, in the mother. The other symptoms are not changed. Cont. hydr. oxym. ex decoct. sarsæ.

The iritis in the child has rather increased.

Hirudines tres oculo affecto. Cont. hydr. cum creta.

*June 4th.* The preceding means have been pursued to this date both for the mother and child. The symptoms in each are completely removed, and they are discharged. They have since been seen, perfectly well.

---

ERYSIPELAS PHLEGMONODES OF THE HAND AND ARM, FOLLOWING THE AMPUTATION OF A FINGER.

James Cossan, æt. 67; was admitted into Kenton's ward, May 10th, under the care of Mr. Lawrence.

The patient, a thin, and rather sallow man, came into the hospital for a foul ulceration of the fore-finger of the right hand, which was of three months standing, and had completely destroyed the last joint of the finger. The greater part of the finger being diseased, Mr. L. determined to remove the whole of it, together with the head of the metacarpal bone. His bowels having been previously regulated, &c. the operation was performed, May 12th.

*14th.* The whole hand is inflamed and swollen, and the inflammation extends a short distance up the fore-arm; the pain arising from it was last night so great as to deprive him of rest; the tongue is coated with a white fur, the bowels are constipated, and he is feverish. On removing the dressings, the flaps were found to have united by adhesion, except at the points where the ends of the ligatures came out.

Hydr. submuriatis, gr. iv. jalapæ, gr. xij. statim. Misturæ sennæ comp. ℥iss. cras mane. Misturæ ammoniæ acet. ℥iss. 6ta quaque hora. Hirudines xij. parti affectæ.

The hand and fore-arm to be afterwards fomented and then covered with a linseed poultice.

17*th*. The inflammation has gradually increased: the whole of the fore-arm is now considerably swollen, and extremely painful. His bowels are well regulated by the mistur. sennæ comp.

V. S. ad ℥xvj. ex brachio affecto.

The blood was very much cupped and buffed.

18*th*. The swelling and inflammation continue gradually to increase.

Hirudines xxiv.—Fomentatio et cataplasma panis.

19*th*. The inflammation of the hand subsiding, but is extending up the arm. Pergat.

20*th*. The inflammation gradually leaving the hand and extending up the arm: the parts in the immediate neighbourhood of the elbow are much swollen, of a bright red colour, very tense, and extremely painful.

Hirudines xxiv.

21*st*. The leeches caused a slight degree of sickness with great weakness. The swelling and inflammation have almost entirely left the hand; about the elbow it has increased, and the greater part of the arm is now much swollen and of a bright red colour: there is a small formation of matter over the olecranon. Mr. Lawrence made a longitudinal incision about four inches in length, through the skin and cellular membrane over the olecranon: a small quantity of pus mixed with blood came away. His pulse is very weak and he is extremely feeble.

Quininæ sulph. gr. ij. ex infus. rosæ, ℥iss. ter in die. Vini rubri, ℥vj. quotidie. Cont. fomentatio et cataplasma.

22*nd*. He took only a portion of the wine yesterday as it made him thirsty and feverish. The inflammation has reached the shoulder: the whole arm very much swollen, tense, and of a bright red colour. Mr. L. made a longitudinal incision through the skin and cellular membrane about six inches long on the posterior part of the arm, and another about three inches in length over the head of the radius; no pus was discharged, but the sides of the incisions presented an unhealthy appearance, as if in an incipient state of suppuration. About six ounces of blood were lost by the incisions.

23*rd*. The tension has been removed by the incisions, and the pain is considerably less. The inflammation has not extended. He took the whole of his wine yesterday.

25*th*. A small formation of matter exists at the upper and inner side of the arm: an incision made into it gave vent to about two ounces of pus. The swelling and inflammation are gradually subsiding: his pulse is more full, softer, and slower. He continues to take the wine and quinine, which agree with him.

27*th*. Continues gradually to improve: the discharge from the incisions is very great.

29*th*. Some sloughing of the cellular membrane in the situation where the last incisions were made: the discharge from them increases in quantity. There is also a little sloughing of the skin at the upper and inner part of the arm. The incisions to be dressed with the ung. resinæ; the poultices to be continued over the dressings. A roller to be applied to the hand and fore-arm.

*June* 1*st*. Complains of great pain in the incisions. The ung. resinæ to be discontinued, and simple dressings used: the poultices to be continued.

4*th*. There is extensive sloughing of the cellular membrane exposed by the incisions; large dirty-looking ragged shreds of it have come away, and left the neighbouring skin undermined. The integuments at the posterior part of the arm are completely separated from the muscles. There is very little sloughing of the skin, and that in one place only—at the upper and inner side of the arm. Let the wine be increased to ℥xij. daily.

8*th*. All symptoms of inflammation have disappeared; the poultices to be discontinued, simple dressings used, and the whole arm rolled.

The above means were continued, until the 29th of July, during which time, he slowly, but progressively improved: the incisions are healed, his health is good, and he is discharged.

*Remarks.* Mr. Lawrence considered this a well-marked case of phlegmonous erysipelas; that is, of erysipelas affecting both the skin and cellular membrane. He mentioned that the most direct and decisive means of relieving tension, preventing extensive sloughing, and checking the progress of the affection, in these cases, consisted in the treatment recommended by Mr. Copeland Hutchison; that of making free incisions through the inflamed parts at an early period of the disease, of which he said that repeated experience had shewn him the great advantages. He also observed, that the same treatment was very advantageous after the processes of suppuration and sloughing had commenced, as preventing the further progress of mischief.

A fact in the above case is certainly worthy of recital, as strongly supporting the doctrine of early incisions in these cases. At the back of the arm, and over the olecranon, the inflammation was greater than in any other part: here an incision was made early, and no sloughing of the cellular membrane took place; while at the upper part of the arm, where the inflammation was much less, and where incisions were not made until a later period, the sloughing process proceeded to an extent which completely undermined the integuments.

---

ERYSIPELAS ŒDEMATODES.

Joseph Henderson, æt. 34, a linen-draper, and a stout, healthy-looking man, was admitted, July 4th, under the care of Mr. Earle. He reports that he has been in the habit of living temperately, and that, six days ago, when in a state of intoxication, he was knocked down, and, in his fall, received a bruise of the integuments, immediately over the tubercle of the tibia, on the right leg. This was on Thursday evening. On the following Monday he walked twenty miles, and, on the same night, first perceived a stiffness in the bruised leg, which had considerably increased in size, from the foot to the hip, by the following morning. It continued gradually to enlarge until Wednesday evening, when he was brought to the hospital. During this time, he had no constitutional disturbance.

*July 4th. 7 o'Clock, p.m.* The whole extremity, from the foot to the hip, is swollen to twice its original size, as may be judged from the appearance of the opposite limb; the leg and foot present the smooth and shining appearance described by Dr. Bateman, but the thigh has a dull appearance, and communicates a roughness to the touch; the whole is of a pale, reddish-brown colour, without tension, and leaves a pit when pressed upon. An abrasion exists in the situation where the bruise was received, and the surrounding integuments, for a small space, are ecchymosed. The local pain is rather severe; the tongue white and moist; pulse small, hard, and 120; the secretions from the skin, bowels, and kidneys, not diminished.

Hydrargyri submur. pulveris antimonialis ana, gr. iij. hora somni sumend. Fomentatio papav. parti affectæ.

*5th.* The extremity appears less swollen, and a few small vesications are observed upon some parts of it. In other respects, its appearances are much the same. The local pain has not been such as materially to disturb his rest. Pulse the same as yesterday; tongue white and moist.

12 *o'Clock, Meridie.* Mr. Earle saw the patient, who descsibes himself as better than he was last night.

Hirudines xl. membro. Hydr. submuriatis, gr. iij. Antimonii tart. gr. ½, hora somni sumend. Cont. fotus.

*6th.* The leeches bled freely; the bowels were twice moved yesterday. He has had much pain in the limb during the night, but it has altogether ceased this morning. The appearances of the limb are not sensiby changed. The pulse has received a marked change; it is now small, frequent, irregular, and easily compressed.

Magnesiæ sulphatis, ʒj. Misturæ ammoniæ acetatis, ℥iss. ter in die. Repr. hydr. submur. et ant. hac nocte.

Let a bread poultice be applied to the bruised surface, and the liquor. ammoniæ acet. to the remaining portion of the limb.

*7th. 8 o'Clock, a.m.* Has been delirious during the night. The erysipelas has extended up the abdomen, and is bounded anteriorly and superiorly by a line drawn two inches above Poupart's ligament, and posteriorly and superiorly by the crista of the ilium; the vesications have increased; the integuments, to the extent of four or five inches surrounding the bruise, are livid and abraded. In other respects, the external characters of the extremity are not changed. The countenance is pale and depicts distress; if he attempt to speak, he can only do it with a stammer. The tongue is dry, and covered with a thick brown coat; the skin, more particularly of the hands and feet, cold and clammy; pulse 130, very small and feeble; the bowels have been twice opened by a dose of castor oil, administered early this morning; stools light-coloured, and very offensive.

Quininæ sulphatis, gr. ij. ter in die. Vini rubri, ℥viij. quotidie.

1 *o'Clock, p.m.* An incision was made by Mr. Earle, from the trochanter major to the external condyle of the femur, and two others, one on each side of the leg in its whole length, through the skin and cellular substance, to the muscles. The cellular tissue of the divided surfaces presented a dark and sloughy appearance, and was of an unnatural firmness. A little bloody serum escaped.

10 *o'Clock, p.m.* Continues delirious; wants to get out of bed, and is obliged to be confined by the waistcoat.

*8th.* 8 *o'Clock, a.m.* Has been raving greater part of the night, but is greatly exhausted this morning; his countenance has a ghastly appearance; his pulse scarcely perceptible; and his teeth and lips coated with a black sordes.

He gradually sunk, and expired at 11 this evening.

*Sectio Cadaveris.* The subcutaneous cellular substance of the leg had completely sloughed throughout its whole extent; that of the thigh was of a dark grey colour, evidently denoting a rapid approach to the same state. It was, however, particularly observed, that it had not made the same progress in the parts corresponding to the vesications; it was here thickened, of a light grey colour, and contained a mixture of pus and serum. The fasciæ of the leg and thigh had a dark appearance, and were not adherent to the muscles. Nothing of importance was observed in the situation of the original bruise.

The dura mater presented a healthy appearance; the arachnoid membrane was rather opaque, in consequence of a slight effusion which had taken place into the texture of the pia mater. The medullary substance of the brain was firmer than natural, and sections of it discovered an unusual number of red points, marking the enlargement of blood-vessels. The lateral ventricles contained an increased quantity of fluid.

The lungs were perfectly healthy. The right cavities of the heart, together with the left auricle, were healthy; the parietes of the left ventricle were very much thickened, and its cavity proportionally diminished in size; the valves were perfectly healthy. The aorta, to the extent of two inches after its origin, was partially lined by strata of effused lymph, which were readily detached, leaving its cuticular or inner coat in a natural state.

A small portion of the mucous membrane of the stomach, about three inches from the pyloric orifice, was of a chocolate colour. The lower two inches of the ileum were very vascular, and there was some degree of congestion about the abdominal viscera generally.

---

The following cases of syphilitic iritis were admitted, under the care of Mr. Lawrence. They are introduced to show the plan of treatment adopted by that gentleman, and the degree of success which invariably attends it when employed in the early stages of the disease.

*Case* 1. Anne Cornwall, æt. 19, of a full habit, florid complexion, and having naturally blue irides, was admitted into Patience ward, on the 1st of June, with syphilitic iritis of both eyes; papular eruption over the body, and superficial ulceration of the tonsils.

Five months ago, she had gonorrhœa, succeeded by an eruption on the forehead, shoulders, and extremities. In two months from the period of admission, she was discharged, having no remains of the disease.

The gonorrhœa returned.

*June* 1*st.* A slight vaginal discharge, together with an eruption of small papulæ over the body, and slight superficial ulceration of the tonsils, were discovered. A fortnight ago her eyes became red, painful, and watering; she had pain in the back of the head and across the brows, which was most severe at night. The conjunctival vessels in each eye are partially injected with blood; those of the sclerotica are greatly distended, and form a pink zone around the cornea. A large and distinct mass of lymph, of a rusty, or reddish-brown colour, covers about one-third of each iris; the rest of its surface having assumed a dull and muddy appearance; the inner circles of the irides are of a rusty-brown colour, and their pupillary margins thickened; the pupils are very much contracted, and fixed in the centre of the irides; the corneæ are hazy, and the eyes have a very inanimate appearance; there is slight intolerance of light, and increased lachrymal discharge. She has little pain, except at night, when it is severe. Vision is very dim; it is with much difficulty she distinguishes large objects; the pulse is increased in frequency; the tongue foul.

C.C. ad ℥xvj. temporibus. Hydr. submur. gr. iij. Jalapæ, gr. x. statim. Cal. gr. ij. Opii, gr. ⅓, 6ta quaque hora. Moistened extract of belladonna to the brows for an hour night and morning.

2*d*. Considerable pain last night in the head and eyes; no alteration in other respects.

Hirudines xx. oculis.

4*th*. Mouth a little affected; the lymph is diminished, and the pain nearly gone.

Hirudines xviij. temporibus.

5*th*. Mouth very sore; great improvement in every respect.

Intr. cal. et opium.

6*th*. Lymph completely absorbed; redness gone; natural colour and appearance of the irides restored; vision perfect; pupils largely dilated by the belladonna; so that, if adhesions had previously existed, which is very probable, they must have been absorbed. The throat is well, and the eruption much better.

Hydr. submur. gr. ij. Opii, gr. ⅓, omni nocte. Discontinue the belladonna.

*July* 2*d*. The eruption has gradually declined, the vaginal discharge ceased, and she is now quite well. Discharged.

*Case* 2. Daniel Burn, æt. 36; *June* 29*th*.

This patient, who has naturally blue irides, was admitted into the hospital, about seven weeks ago, with an indurated chancre on the back of the prepuce, and suppurating bubo in the left groin. In a fortnight the chancre healed, whilst under the influence of mercury; the bubo was opened, and discharged a quantity of pus; and, in a day or two afterwards, he was made an out-patient. From that time to the present he has taken diet-drink only.

*June* 27*th*. He came to the hospital, complaining of pain in the right eye, and slight intolerance of light. Upon examination, a pale zone, of a pink or rose colour, was observed around the cornea, in the sclerotic coat; the iris a little discoloured, the pupil contracted, and vision somewhat indistinct.

Mr. Lawrence advised him to come into the hospital; he refused, and was, therefore, ordered—

Hydr. submuriatis, gr. ij. opii, gr. ⅓, sexta quaque hora.

*June* 29*th*. The eye continued to get worse; he again applied at the hospital and was this day admitted.

A discolouration and thickening of the prepuce mark the situation of the original sore.

The conjunctiva is reddened. A broad zone of vessels, of a pink or rose colour, is situated around the cornea, in the sclerotic coat. The iris presents a dull, yellowish green appearance, and has lost its motions. The pupil is much contracted, but preserves its circular form. The cornea is hazy. Vision is much impaired; he cannot read large print. He has great pain over the eyebrow, and in the back of the head, which he describes as very severe in the evening and night, but remitting in the morning.

No other constitutional symptom has made its appearance.

C. C. ad ℥xiv. temp. dextro. hydr. submur. gr. ij. opii, gr. ⅓, sexta quaque hora. Mist. sennæ c. pro r. n.

30*th*. The eye much easier; mouth a little affected. Pergat.

*July* 1*st*. A degree of uneasiness coming on in the eye last night, he was again cupped to ℥xiv. from the right temple. The mouth is a little affected. He feels easier. Vision is more distinct.

Cont. cal. et opium.

2*nd*. Mouth very sore. Vision continues to improve.

Hydrargyri submur. gr. ij. opii, gr. ⅓, octavis horis. Moistened extract of belladonna to the eyebrow for an hour night and morning.

*3rd.* Much the same as yesterday. Pupil a little dilated by the belladonna.

*4th.* The external redness is diminished; the unnatural colour of the iris partially removed. He complains of a nocturnal pain over the brow.

Ung. hydrargyri, gr. vj. opii (redacti in pulverem subtilem), gr. ij. ft. Ung. regioni supercilii applicandum hac nocte. Extr. belladonnæ omni mane.

*5th.* Not the slightest recurrence of pain in the brow last night.

*7th.* The mouth continues sore. The eye has resumed its natural appearance. Vision is perfect. The pupil has been dilated by the belladonna; the pupillary margin presents a very irregular appearance in consequence of the adhesions subsisting between its posterior surface and the capsule of the lens.

*9th.* Made an out-patient.

*Remarks.* Mr. Lawrence considered the former case a most beautiful specimen of syphilitic iritis; and remarked that, whenever the effused lymph presented the appearance which it assumed in that instance, we might at once decide upon the nature of the disease. Nothing but the previous history of the patient could have enabled us to distinguish the latter case from the idiopathic or gouty varieties of the disease.

---

### PUNCTURE OF THE BLADDER ABOVE THE PUBES.

John Dainton, æt. 48, was admitted on the 21st of June, with retention of urine. The patient was in the hospital about a year since with stricture, from which he has suffered nine years. On Sunday last (June 17th), about four o'clock in the afternoon, he made a little water, but with some degree of difficulty and pain. From that time to the present (June 21st), he has not voided any urine. On the 19th he went to a surgeon, who directed that eight leeches should be applied to the perineum, and endeavoured to pass a catheter, but without success. On the 20th he was bled to ℥xxiv. and had cloths, wetted with warm water, applied to the perineum.

*At half-past* 11, *a. m.* On the day above-mentioned, he was admitted into the hospital, when he complained of very acute pain in the abdomen; the bladder was greatly distended; his pulse quick, hard, and full; tongue dry; skin hot. A warm bath was immediately ordered, after which Mr. Lawrence attempted the introduction of a catheter, but failed. He was then ordered

V. S. ad ℥xviij. hydr. submur. gr. iv. jalapæ, gr. xij. statim sumend. et, post horas tres, misturæ sennæ comp. ℥iss.

*Five o'clock, p. m.* The warm bath was repeated; from which he received relief, and made a few drops of urine, and had also two evacuations from his bowels.

*Half-past* 9, *p. m.* He was again bled to ℥xvj. had another warm-bath, and was ordered to take ʒvj. of liq. antim. tartarizati, every quarter of an hour, for four times.

These remedies procured great ease, and a quantity of urine passed, which wet his shirt and the bed-clothes considerably.

*Quarter-past* 10, *p. m.* He had just left the bath, and was in a profuse perspiration, when Mr. Lawrence saw him, who directed that the same treatment should be persevered in, if necessary, with the exception that the six-drachm doses of the liquor antim. tart. should be discontinued, and one drachm administered every four hours.

*June 22nd,* 8 *a. m.* Has passed a tolerable night, and voided urine in small quantities at different periods. Feels comfortable this morning. Pulse quick and hard.

V. S. ad ℥xij. Rep. balneum.

*Eleven, p. m.* Felt relief from the bath, and made a little water whilst in it. There is considerable tension of the abdomen, and slight pain on pressure; bowels not open.

Hydr. submur. gr. iv. jalapæ, gr. xij. statim. Misturæ sennæ c. ℥iss. et rep. 4ta quaque hora donec alvus plenè resp.

*23rd.* The bladder appears more distended. The pain experienced upon pressure is greater than at any former period. Has passed urine in a small quantity during the night. Bowels open; pulse quick, but neither hard nor full. (Rep. balneum.) Mr. L. attempted to pass a catheter, but could not succeed.

Magnes. sulph. ℨj. mist. ammon. acet. 6ta quaque h.

*Three, p. m.* The bladder appearing more distended than heretofore (the fundus apparently being higher than the umbilicus), and the pain upon pressure continuing to increase, Mr. L. thought it proper to puncture the bladder above the pubes. The trocar was introduced about an inch and a half above the pubes, in a direction obliquely downwards, and, when withdrawn, about a pint of very high-coloured urine escaped. An elastic gum catheter, with a bladder affixed to its extremity, was then introduced through the canula, and the latter withdrawn. Great relief followed the operation.

*24th.* No urine has been collected in the bladder. During the night urine has several times passed, in small quantities, through the urethra.

*25th.* Has passed a bad night; complains of pain in the abdomen. No urine has passed through the catheter into the bladder attached to it; but the patient has, with great pain, voided some through the urethra. The catheter was withdrawn.

Mist. sennæ c. p. r. n. Cont. mist. salin. cu. ant. et magnes. sulph.

*26th.* Has not slept during the night; complains of great pain upon pressing the abdomen; bladder appears greatly distended, and presses much against the rectum; passed some urine through the urethra during the night.

As no catheter could be passed into the bladder through the urethra, Mr. Lawrence thought it advisable to puncture the bladder again above the pubes; which he did, first making an incision through the parietes with a scalpel. On withdrawing the trocar no urine followed. The edges of the wound were drawn together with adhesive plaster.

*Two, p. m.* Mr. Lawrence wished to make an opening into the bladder by dividing the stricture from the perineum; but the patient being too weak, he did not perform the operation.

*Eleven, p. m.* Great pain in the abdomen upon the least pressure; very feeble pulse.

Hirudines xxiv. abdomini, et postea fotus tepidi.

*27th.* Bowels not open during the night; great pain on pressure; skin very hot and dry; tongue much furred. Says he made water in a small stream during the night.

Olei ricini, ℥j. statim sumend.

*Eleven, p. m.* Has been sleeping all the evening; does not complain so much of pain upon pressure; has passed urine in a tolerable stream during the day.

Cont. medicamenta.

*28th.* Bowels open; pain upon pressure subsiding; has passed urine through the urethra; discharge of healthy pus from the wound.

*July 16th.* The patient has been gradually improving since the last date; he has continued to make water through the natural passage, in a small stream, and that without pain or difficulty; the wound has very nearly healed, and he finds no inconvenience from it. It was the wish of Mr. Lawrence that the patient should remain in the Hospital, and have his urethra attended to, but he chose to leave on this day.

---

### ANEURISM OF THE LEFT FEMORAL ARTERY AT THE MIDDLE OF THE THIGH.

Daniel Callaghan, æt. 42, was admitted June 6th. The patient is a bookbinder; has a sallow unhealthy countenance, with some redness of the nose, and a full, strong pulse. He does not trace his complaint to any external or local cause. Having been previously in good health, he had an attack, a fortnight before Christmas, of what was considered inflammation of the liver, for which antiphlogistic measures were resorted to with success. He noticed the swelling in his thigh about four or five months ago; it was then smaller than at present, and free from pain. The tumour, not having been properly examined, was mistaken for an abscess, and treated accordingly. There was slight swelling and numbness of the limb.

The tumour, which is about two inches in diameter, and pulsates strongly, is situated in the course of the femoral artery, just above its passage through the triceps. It is firm to the feel, becoming less and softer when the influx of blood is prevented by pressure on the femoral artery above. There is pain in the swelling, but no change of colour. The femoral artery, at the crural arch, feels as large as the fore-finger, and pulsates very powerfully. The anterior and posterior tibial arteries can be felt at the foot, but not so plainly as on the other side. Slight œdema and numbness of the limb: the veins rather fuller than on the other side. Pulse full, frequent, and hard; tongue furred; skin hot.

Hydr. submur. gr. iv. jalapæ, gr. xv. statim. Mist. sennæ c. cras mane.

*June 7th.* The bowels have been freely opened; symptoms but little altered.

V. S. ad ℥xx. misturæ ammon. acet. ℥iss. 6tâ quâque horâ.

*8th.* Febrile symptoms diminished; pulse still hard.

Hydr. submur. gr. iv. jalapæ, gr. xv. statim et mist. sennæ c. ℥iss. postea. Lotio Goulard. tumori.

*9th.* The bowels have been freely opened; tongue cleaner; pulse 82 and hard.

*Half-past* 12 *meridie.* The artery was tied by Mr. L. with a single ligature on the inner edge of the sartorius. The pulsation in the tumour and arteries below it immediately ceased.

*Nine* P. M. The temperature of the left, or affected limb is higher than that of the opposite side. Pulse full, hard, and frequent; skin hot and dry.

V. S. ad ℥xiv.

*10th.* Arterial action, though diminished, still high; the bowels are not sufficiently open.

Hydr. submur. gr. iv. jalapæ, gr. xij. cum infus. sennæ c. ℥iss. statim.

*Nine o'clock,* P. M. The bowels not having acted, and the vascular excitement continuing unreduced, he was again bled to ℥xij. when copious stools were obtained.

Yesterday morning, for the first time, and previous to the operation, a small pulsating tumour, about the size of a hazel-nut, and situated rather higher in the limb, was observed in the other thigh.

*11th.* Pulse reduced; bowels freely open. The wound was opened and dressed, and its edges found, in great part, to be united.

*13th.* The patient is going on favourably; pulse natural, with the exception that there is a little inclination to hardness; wound looking well.

*20th.* The ligature came away to-day. Wound looking well; pulse natural; bowels open.

*23rd.* One side of the wound is red, painful, and rather hard: the case is otherwise doing well. (Cataplasma lini.)

*27th.* The wound is nearly healed; the discharge from it slight. (Let common dressings be applied.)

*July 2nd.* Some inflammation and hardness about the wound. Hirudines xij. et postea, cataplasma lini.

*5th.* Inflammation, with redness, swelling, induration and great pain have taken place in the thigh, below and on the inside of the wound. The soft parts, underneath the wound, are occupied by an inflammatory induration, which forms a hard lump, equal in size to an orange, under the skin. The pulse is accelerated, the tongue foul, the skin hot and dry.

V. S. ad ℥xiv. hirudines xviij. parti affectæ, cont. cataplasm. hydr. sub. gr iv. jalapæ, gr. x. statim.

*8th.* The inflammation is much reduced; the discharge from the wound increased, the hardness underneath remains.

Mistur. sennæ c. pro re nata; cont. cataplasma.

The aneurismal tumour in the right thigh remained stationary. He continued to mend, and was able to walk very well with a crutch, when he left the hospital, without leave, on the 29th of July.

---

### MORBID ALTERATION OF STRUCTURE IN THE SYNOVIAL MEMBRANE OF THE KNEE.

*Case* 1. John Barnet, æt 25; was admitted into the hospital on the 8th of May, under the care of Mr. Vincent.

He says that his knee first became affected two years ago and without any evident cause. It commenced with a stiffness, which continued, and was always greater after quietude, but after walking a short distance it subsided, and the limb regained its original mobility. It continued in this state for six or eight months, when he perceived a slight degree of swelling in it, but was still able to pursue his accustomed avocations. He could walk, although with some degree of pain at starting, and always suffered much when he next became quiet. Three months before admission his health became affected, and the pain produced by motion was such as obliged him to keep the limb in a state of perfect quietude. He had taken medicines, applied leeches and blisters, but with only temporary benefit.

*May 8th.* The joint is about three inches greater in circumference than the opposite one; the swelling is equable, and presents above and on either side of the patella a soft and elastic substance; its temperature is higher than that of the opposite joint. Says he is very weak and has lost flesh, which he refers to want of rest, occasioned by the pain which he suffers. Pulse small and frequent, tongue white, bowels regular.

C. C. ad ℥viij. genu, pilulæ saponis cum opii, gr. v. omni nocte, mistura sennæ comp. pro re nata.

When the limb gets into a quiet state, let a blister be applied.

*17th.* An absces which pointed on the inside of the tibia was opened, and a small quantity of pus discharged.

*June 16th.* He has got weaker and weaker; his pulse is small and frequent, and he continues to lose flesh. The cupping and blister gave a temporary relief. The anodyne medicines have failed to procure rest, although the dose has been increased; and if they occasion him to doze, he is soon disturbed by the jumpings of the limb.

It was judged necessary to amputate the leg; which was this day done by Mr. Vincent, who in this as in the generality of his cases, preferred the double flap operation. After the first incisions had been made, the bone sawn through, and the femoral artery with two or three smaller vessels tied; the flaps were approximated by means of adhesive plaster, a compress of lint placed on either side, which was secured by a roller applied from above downwards, and the patient carried to bed.

In two hours hæmorrhage took place, when it was found necessary to open the stump and secure two other small vessels. Damp cloths were applied for a few hours, when, all appearances of hæmorrhage having subsided, the stump was again put up.

*19th.* The stump was opened, and the greater part of the wound found to be united. Simple dressings and the roller were directed to be continued.

*24th.* Ligatures came away. Wound perfectly healthy.

*July 1st.* In consequence of a little disturbance to his general health, the stump has presented an unhealthy appearance, but is now proceeding favourably.

*Aug. 3rd.* The wound has healed; the patient has a very good stump, and he was this day discharged.

*Examination of the Joint.*—The synovial membrane, except where it was reflected over the cartilages, was found converted into the pulpy, and yellowish brown substance, intersected by white lines, described by Mr. Brodie (see plate.) The cartilages covering the condyles of the femur, and corresponding articulating surfaces of the tibia were ulcerated at a few points, and presented generally a rough surface. The crucial, external, and internal lateral ligaments, and the semilunar cartilages retained their natural structure. The abscess did not communicate with, neither was there any unnatural fluid in the joint.

*Case* 2. Joseph Collins, æt. 18; a blacksmith, was admitted into Luke's ward on the 16th of June, under the care of Mr. Vincent, and gives the following history of his case.

Three years ago a cricket-ball struck the left knee, which was followed by slight occasional pain, and little or no swelling of the joint. It continued in this state for the two first years, the symptoms being alternately aggravated and lessened, by motion and quietude. Up to this period no remedies were employed for his relief. About ten months previous to admission, two blisters were applied, and from that time he describes the pain as having been very acute.

*June 16th.* The knee is slightly increased in size; not however from any effusion into the joint, as made known by the absence of fluctuation, but by a morbid alteration of structure in the synovial membrane, as manifested by the peculiar elastic feel characteristic of that disease. The parts external to the joint appear thickened in consequence of chronic inflammation. He refers the pain which he suffers to the inside of the knee, extending from thence along the tibia to the foot, and describes it as excruciating during the night, occasioning him to start if he doze. If the patella or sole of the foot be pressed towards the articulation the greatest pain is produced. He generally keeps the limb resting on its outer side, and in a half flexed position. He looks tolerably healthy, yet complains of great weakness; pulse small and frequent; tongue clean.

A broth diet was ordered. It was directed that leeches should be applied and aperient medicines administered to bring the joint into a quiet state, and then a caustic issue made on its inner side; as also that the pilula saponis cum opio should be given every night, and the bowels regulated by occasional doses of house physic.

*July 6th.* The preceding remedies have been employed with merely temporary benefit. Although the dose has been increased the anodynes have failed to produce their wonted effects. The pain he suffers is very severe, his strength is declining, and he earnestly solicits the removal of his limb.

*7th.* Mr. Vincent removed the limb, as in the former case, by the double flap operation. The vessels being secured, the flaps were approximated by adhesive plaster assisted by a compress on either side, which was secured by a roller passed from above downwards. The patient was carried to bed, a pillow placed underneath the stump, and damp cloths applied over it.

10*th.* Dressings removed. Greater part of the wound united. Gets more rest than he has done for a long time previously.

15*th.* Ligatures came away. Wound looks perfectly healthy.

*August* 14*th.* The stump has been completely healed a fortnight since, but the patient remains in the hospital for the completion of his wooden leg.

*Examination of the Joint.* The external parts were in some measure thickened. The external and internal lateral ligaments preserved their natural structure. No traces of natural organization could be discovered in the synovial membrane except where it was reflected over the cartilages; it had been converted into that thick, pulpy substance, of a reddish brown colour and intersected by white membranous lines, described by Mr. Brodie, and which, as the same gentleman has remarked, is peculiar to it. The crucial ligaments were thinner than natural: the semi-lunar cartilages entire; those covering the condyles of the femur and corresponding articular surfaces of the tibia, were not more than ¼ of their ordinary thickness, presented a roughness to the touch, and were very readily separated from the bones, the exposed surfaces of which were very vascular, and softer than cartilage in its natural state.

*Remarks.* The two preceding cases were beautiful specimens of the morbid alteration of structure in the synovial membrane, described by Mr. Brodie in the third chapter of his valuable work on "Diseases of the Joints." Nothing but the peculiar elastic feel, which the application of both hands to the joint distinguished from fluctuation, could possibly have predicted the nature of the latter case; the symptoms, throughout its whole progress, being exactly similar to those produced by ulceration of the cartilages occurring as the primary disease. The vascularity and softness of the articulating extremities of the bones, as demonstrated after the removal of the cartilages, were probably connected with a state of the bone preceding the commencement of caries;* as the cartilages covering them were not, at some points, thicker than a wafer. They could not, I think, be attributed to any scrofulous affection going on in the bones; for a modern author† has observed, that, in the latter disease, he has found the vascularity diminished in proportion to its duration. The cancellous structure was also healthy, and perfectly free from any caseous deposit.

---

### OSTEO-SARCOMATOUS TUMOUR OF THE LOWER JAW.

Henry Roberts, æt. 61; was admitted July 4th, under the care of Mr. Lawrence.

The patient has been subject to slight

---

* I use the term as denoting that state of bone which corresponds to ulceration in soft parts.

† Lloyd, page 128.

rheumatic attacks; in the intervals of which he has enjoyed good health.

About fourteen years since a small vascular tumour, increasing in size very slowly and attended with very little pain, made its appearance on the alveolar process of the lower jaw, in front of the incisor teeth. About two years and a half ago, when it had attained the size of a walnut, it was removed by the knife. Shortly afterwards, it again began to grow, proceeded more rapidly than before, was attended with much pain, and projected both in front and behind the teeth.

The tumour now occupies the alveolar process and the body of the bone from its middle to the first molar tooth, and consists of two portions, one of which occupies the situation of the gum, while the other rests firmly on the bone. The former is a red and vascular excrescence, softish and movable; while the latter, covered by the mucous membrane of the mouth, is white, cartilaginous in texture, and identified with the bone. The teeth are seated in a furrow of the vascular excrescence, and quite loose.

As the tumour descended nearly to the basis of the jaw, Mr. Lawrence thought it advisable to remove entirely that portion of the bone in which the morbid growth was situated, as being the only effectual method of preventing a relapse of the disease; the patient, however, would not submit to that proceeding, but wished that the diseased parts should be taken away.

The loose teeth having been previously removed, the operation was performed *July 13th.* The vascular fungous excrescence and its cartilaginous basis were completely dissected away, leaving a semicircular gap in the bone, extending nearly to its basis. In this part the substance of the bone had been converted into a tumour of cartilaginous consistence with numerous small bony spicula projecting into it.

*19th.* The patient is altogether free from pain and constitutional disturbance. The wound has a perfectly healthy character.

*22nd.* Was made an out-patient, the wound still retaining its healthy appearance, and being considerably reduced in size.

*August 10th.* Came to the hospital. The wound was healed, and the parts, lately occupied by the tumour, were perfectly healthy.

---

CANCER SCROTI.

James Dean, æt. 23; came to the hospital on the 9th of August, with a carcinomatous ulceration of the scrotum; commonly known by the name of chimney-sweepers' cancer; or soot wart.

Four years ago, the patient, who is a sweep, observed an indurated pimple, about the size of a small pea, on the lower part of the scrotum, which remained stationary for two years, when he removed the top and a sore formed, which has continued to enlarge. Its progress has been much more rapid during the last six months, and, until within the last six weeks, the pain has been trifling.

A tuberculated induration, half the size of the first, and detached from the subjacent parts, occupies the whole of the lower part and right side of the scrotum. Upon the centre of this is an irregular ulcerated surface which secretes a small quantity of a thin sanies, and is surrounded by hard, ragged, and unequal edges. The local pain is severe, and such as prevents his resting at night; yet his constitution is not affected by it.

Mistura sennæ comp. pro re nata.

*August 11th.* Mr. Earle dissected away the morbid part, and exposed the tunica vaginalis and testicle, on each side, in a perfectly healthy state. Two or three small pimples, similar to that which originally produced the disease, were observed on the neighbouring part of the scrotum, and removed. It had been necessary to take away so large a portion of the scrotum, that the remaining part was not sufficient to cover the testicles: they were therefore left, together with the corresponding tunica vaginalis, bare. The patient was then put into bed, a dose of laudanum administered, a pillow placed to support the testicles, and directions given that the parts should be kept damp with cold rags. 8 *o'clock* P.M. ol. ricini, ℥j. statim.

*13th.* Has slept well during the two last nights. Complains of slight pain in the testicles. Bowels well open—tongue clean.

Catapl. panis. Cont. mistura sennæ c. pro re nata.

*20th.* The patient has experienced a slight attack of hernia humoralis; a stratum of lymph has been effused over the bare surface; granulations have formed in it, and the part presents a perfectly healthy appearance.

*22nd.* Cicatrization commencing. Is free from pain.

*25th.* The health continues good, and cicatrization is proceeding rapidly.

*27th.* Continued favourable progress.

---

CARCINOMATOUS ULCERATION OF THE LEFT THUMB.

Read Fuller, æt. 55, a farmer's labourer, who had lived regularly, and enjoyed good health, came from the country, and was admitted into the hospital, July 12th.

The patient had had several seedy warts on the left hand for some time, when, about sixteen months since, one of them, situated on the back part of the thumb, inflamed, swelled, and, as he says, "festered off." It discharged a thin watery fluid for a short time, and, being rubbed and irritated in the course of his employment, slowly but progressively increased in size, so that, three months previous to admission, it had attained the size of a large egg; being free from pain, and not ulcerated. By the advice of a neighbouring servant, he applied caustic to the part every day for a month, under the use of which it ulcerated. He now discontinued his employment, and, by the direction of a surgeon to whom he applied, used a wash, during which time the disease grew worse and became painful, and continued to increase until the period of admission.

The case being a very interesting one, I beg to give Mr. Lawrence's description of it.

"The disease now covers the first bone of the thumb, (the first metacarpal bone of some anatomists) and the joint between it and the second phalanx, stopping a little short of the carpal extremity of the radius; it occupies one-half of the ball of the thumb, and reaches nearly to the metacarpal bone of the fore-finger. It consists of a red, hard, uneven, and tuberculated swelling of the skin, partially ulcerated on the surface, and immoveably fixed by a hardened base, on the subjacent parts. There are several excavations in this indurated mass, about equal in size to a sixpence or shilling; some of these are ulcerations, with a clean red surface, but no granulations, eating into the part, and forming rather deep holes, with sharp abrupt edges; others have a fibrous warty character. All of them discharge a thin yellowish clean ichor, with a very peculiar odour. The edge of the mass is tuberculated, and the disease extends by the successive ulceration of the tubercles, which enlarge, become soft, and then form fresh excavations. The integuments are red, hard, and fixed beyond the limits of the active disease. The fore-arm and arm are unaffected, but the axilla presents a glandular enlargement, about equal to a pigeon's egg, rather soft, moveable, free from pain, and without any discolouration of the skin. The patient considers that this swelling has no connexion with the disease of the thumb, and represents that the former has existed for many years. He has a sallow, and rather unhealthy appearance, with a white tongue, and some feverishness, which he ascribes to the fatigue of his journey, stating that he had been previously in good health, although suffering great pain in the part, especially at night."

Ten leeches to be applied round the disease, and afterwards a bread poultice. Hydr. submur. gr. iv. Jalapæ, gr. xij. statim. Misturæ sennæ c. ℥iss. cras mane, et repr. pro re nata.

*14th.* The inflammation and pain attendant upon it are much diminished.

*15th.* Much the same as yesterday.

Repr. hirudines.

*16th.* Health improved. Inflammation and pain diminished.

Hirudines, xij. Repr. cataplasma panis.

*17th.* Very much easier. Tongue clean; no fever.

Misturæ sennæ c. ℥iss. cras mane.

*18th.* Mr. Lawrence, who regarded the case as a well-marked instance of carcinomatous ulceration, and who considered tubercular induration, the peculiar character of the ulcerations, the thin, ichorous, and fetid discharge, and the severe pain, clear proofs of its malignant nature, had determined upon amputating to-day; all who had previously seen the disease, concurring with him in the opinion of its necessity. As, however, one of his colleagues conceived the affection might give way to rest and mild antiphlogistic treatment, the operation was postponed.

Contr. cataplasm. acet.—mistura sennæ comp.

*20th.* Bowels not sufficiently lax.

Aloes, saponis ana, gr. v. omni nocte.

*23rd.* Rep. hirudines xij.

*27th.* No further advantage having resulted from the above treatment, the question of amputation was again considered, when it was suggested that the cinnabar fumigation might be tried with advantage. (Let it be applied night and morning.)

*August 6th.* Violent inflammation, attended with considerable swelling, deep redness, and severe pain have come on in the part, and extend over nearly the whole hand: red lines extend from the inflamed part to the axillary tumour, which has attained twice its original size. The entire fore-arm and arm are very painful, and the swelling in the axilla is in the same state. The patient has headache, thirst, loss of appetite, and a white tongue. (Let the fumigation be immediately discontinued.)

Hirudines xij. parti affectæ. Misturæ ammoniæ acet, ℥iss. sextâ quaque hora. Liquor plumbi subacetatis dilut. brachio et tumori axillæ.

*10th.* Inflammation a little reduced.

Rep. hirudines xij. Cont. pilul. aloes cum sapone et mistura ammoniæ acetatis.

*13th.* Great relief followed the application of the leeches. The feverish symptoms are subsiding.

Rep. hirudines xij. Cont. medicamenta.

*15th.* Continued favourable progress.

Rep. hirudines xij.

*17th.* Rep. hirudines xij. Cont. medicamenta.

*18th.* The part having got into a quiet state, and the patient being free from fever, Mr. Lawrence determined upon removing the disease to-day.

The tourniquet being applied to the brachial artery, and the hand placed in a state between pronation and supination, Mr. L. felt for the styloid processes of the radius and ulna; immediately before the latter of which, on the back of the hand, he introduced a catlin, and pushing it forwards underneath the integuments and tendons until it protruded in front of the styloid process of the radius, cut out in a direction towards the phalanges of the fingers; thus making a semilunar flap of the integuments. In the same manner was made a corresponding flap on the palmar aspect of the wrist; the joint was opened, and the first row of carpal bones detached from their articulation with the scaphoid cavity of the radius. The radial and ulnar arteries having been secured, the flaps were approximated by adhesive plaster, and directions given that the stump should be kept damp with wet cloths.

*20th.* The stump was opened, and the edges of the wound found approximated, except in the situation where the ligatures came out. The arm is easy, and the patient free from fever.

Simple dressing to the stump.

*22nd.* Little redness about the stump, with pain up the arm.

Hydr. submur. gr. iij. jalapæ, gr. xij. statim. Cataplasma panis.

*23rd.* Increased redness, extending a short way up the fore-arm. Slight fever. (Let the poultice be continued to the stump, and Goulard's wash applied to the fore-arm.)

Misturæ sennæ c. ℥iss. statim.

*25th.* Redness and fever gone. Pergat.

*27th.* Going on favourably in every respect. The axillary tumour remains stationary; if there be any change it is slightly diminished.

The hand was conveyed to the Museum to be injected by Mr. Stanley; I had not, therefore, an opportunity of observing its interior.

THOMAS FEREDAY.

